# Emotional Awareness and Expression Therapy vs Cognitive Behavioral Therapy for Chronic Pain in Older Veterans: A Randomized Clinical Trial

**DOI:** 10.1001/jamanetworkopen.2024.15842

**Published:** 2024-06-13

**Authors:** Brandon C. Yarns, Nicholas J. Jackson, Alexander Alas, Rebecca J. Melrose, Mark A. Lumley, David L. Sultzer

**Affiliations:** 1 Department of Mental Health, VA Greater Los Angeles Healthcare System, California; 2 Department of Psychiatry and Biobehavioral Sciences, David Geffen School of Medicine at University of California, Los Angeles; 3 Department of Medicine, David Geffen School of Medicine at University of California, Los Angeles; 4 Department of Psychology, Wayne State University, Detroit, Michigan; 5 Department of Psychiatry and Human Behavior, University of California, Irvine School of Medicine, Irvine

## Abstract

**Importance:**

Chronic pain is common and disabling in older adults, and psychological interventions are indicated. However, the gold standard approach, cognitive-behavioral therapy (CBT), produces only modest benefits, and more powerful options are needed.

**Objectives:**

To evaluate whether emotional awareness and expression therapy (EAET) is superior to CBT for treatment of chronic pain among predominantly male older veterans and whether higher baseline depression, anxiety, or posttraumatic stress disorder (PTSD) symptoms—key targets of EAET—moderate treatment response.

**Design, Setting, and Participants:**

This 2-arm randomized clinical trial was conducted from May 16, 2019, to September 14, 2023, in the US Department of Veterans Affairs Greater Los Angeles Healthcare System. The trial included a racially and ethnically diverse group of veterans aged 60 to 95 years with at least 3 months of musculoskeletal pain.

**Interventions:**

Emotional awareness and expression therapy or CBT, conducted concurrently, each presented as one 90-minute individual session followed by eight 90-minute group sessions.

**Main Outcomes and Measures:**

The primary outcome was Brief Pain Inventory pain severity (range, 0 to 10) from baseline to posttreatment (week 10, primary end point) and 6-month follow-up. Secondary outcomes included Patient Reported Outcomes Institute Measurement System Anxiety, Depression, Fatigue, General Life Satisfaction (NIH Toolbox), Pain Interference, and Sleep Disturbance Short Forms, Patient Global Impression of Change (PGIC), and Satisfaction with Therapy and Therapist Scale-Revised. A subset of participants completed the PTSD Checklist for *Diagnostic and Statistical Manual of Mental Disorders* (Fifth Edition). All analyses were for the intention-to-treat population and included all randomized participants.

**Results:**

Among 126 randomized participants (66 in the EAET group and 60 in the CBT group; mean [SD] age, 71.9 [5.9] years; 116 [92%] male), 111 (88%) completed posttreatment, and 104 (82%) completed the 6-month follow-up. The EAET was superior to CBT for the primary outcome of reduction in pain severity at posttreatment (estimate, −1.59 [95% CI, −2.35 to −0.83]; *P* < .001) and follow-up (estimate, −1.01 [95% CI, −1.78 to −0.24]; *P* = .01). A greater percentage of participants in EAET vs CBT had clinically significant (at least 30%) pain reduction (63% vs 17%; odds ratio, 21.54 [95% CI, 4.66-99.56]; *P* < .001) at posttreatment. In addition, EAET was superior to CBT on 50% pain reduction (35% vs 7%; odds ratio, 11.77 [95% CI, 2.38-58.25]; *P* = .002), anxiety (estimate, −2.49 [95% CI, −4.30 to −0.68]; *P* = .006), depression (estimate, −3.06 [95% CI, −5.88 to −0.25]; *P* = .03), general life satisfaction (estimate, 1.23 [95% CI, 0.36-2.10]; *P* = .005), PTSD symptoms (estimate, −4.39 [95% CI, −8.44 to −0.34]; *P* = .03), PGIC score (estimate, 1.46 [95% CI, 0.77-2.15]; *P* < .001), and global treatment satisfaction (estimate, 0.28 [95% CI, 0.12-0.45]; *P* < .001) at posttreatment. Higher baseline depression (estimate, −1.55 [95% CI, −0.37 to 2.73]; *P* < .001), anxiety (estimate, −1.53 [95% CI, −2.19 to −0.88]; *P* < .001), and PTSD symptoms (estimate, −1.69 [95% CI, −2.96 to −0.42]; *P* = .009) moderated greater reduction in pain severity after EAET but not CBT.

**Conclusions and Relevance:**

The results of this randomized clinical trial suggest that EAET may be a preferred intervention for medically and psychiatrically complex patients with pain. The societal burden of chronic pain could be improved by further incorporating the principles of EAET into mainstream clinical pain medicine.

**Trial Registration:**

ClinicalTrials.gov Identifier: NCT03918642

## Key Points

**Question:**

Is group-based emotional awareness and expression therapy (EAET)—a psychological intervention targeting trauma and emotional processing—superior to cognitive-behavioral therapy (CBT) for treatment of chronic pain in a racially and ethnically diverse cohort of older veterans?

**Findings:**

In this randomized clinical trial with 126 participants, those randomized to EAET had significantly greater improvements in the primary outcome of reduction in pain severity from baseline to the primary end point of posttreatment (week 10). Moreover, 63% of EAET participants had clinically significant (at least 30%) posttreatment pain reduction vs only 17% in CBT.

**Meaning:**

These findings support the superiority of EAET compared with CBT in reducing chronic pain among older veterans.

## Teaser

This randomized clinical trial assesses whether emotional awareness and expression therapy is superior to cognitive-behavioral therapy (CBT) for treatment of chronic musculoskeletal pain among a racially and ethnically diverse cohort of older veterans.

## Introduction

Chronic pain affects many older adults^1,2^ and is a risk factor for cognitive decline^3^ and premature death.^4^ Medical and surgical interventions, particularly opioids, often produce only partial efficacy^5^ and pose substantial risks,^6^ including polypharmacy,^7^ drug-drug interactions,^8^ and falls.^9,10^ Psychological interventions may be safer, but standard options, such as cognitive-behavioral therapy (CBT), provide only small effect size benefits.^11,12^ In addition, veterans have high rates of severe pain^13^ and overlap between pain and psychiatric conditions, such as depression, anxiety, and posttraumatic stress disorder (PTSD),^14,15,16,17^ which may further limit the effectiveness of CBT.^18^

Adverse childhood experiences,^19,20,21^ military combat,^22^ racism or discrimination,^23,24^ and psychiatric conditions^16,25^ are associated with the presence and severity of chronic pain, but are not directly addressed by CBT. Emotional awareness and expression therapy (EAET)^26^ was developed to target these critical drivers of chronic pain.^20,27,28,29,30^ Emotional awareness and expression therapy demonstrated efficacy in several controlled and uncontrolled trials,^31,32,33,34,35,36,37,38,39^ including 2 showing some superiority to CBT.^40,41^ Yet most trials of EAET have been in largely white, female, often young samples, and testing in an older, diverse population is needed. Given preliminary evidence of efficacy of group-based EAET in small samples of diverse older veterans,^39,41^ the current full-scale trial evaluated whether group-based EAET is superior to CBT among a racially and ethnically diverse cohort of older veterans on the primary outcome of pain severity, and on secondary or exploratory mood and functional outcomes, from baseline to the primary end point of posttreatment (week 10) and to 6 months posttreatment. The effects of baseline levels of depression, anxiety, and PTSD symptoms on extent of pain reduction were also assessed.

## Methods

### Study Design

This randomized clinical trial was conducted at the US Department of Veterans Affairs (VA) Greater Los Angeles Healthcare System (GLA) to evaluate comparative efficacy and moderators of EAET and CBT (trial protocol in Supplement 1). Ethics approval was provided by the institutional review board at GLA, and the trial was preregistered. Enrollment occurred from May 16, 2019, to February 1, 2023, with the final follow-up on September 14, 2023. The study was paused because of COVID-19–related administrative holds from March 20, 2020, until February 4, 2021, at which time a small number of patients who had provided informed consent but had not been randomly allocated to a group were transitioned to a separate single-arm trial of EAET presented via video telehealth.^39^ The study was monitored by the VA Clinical Science Research and Development Data Monitoring Committee. All participants provided written informed consent. This report followed the Consolidated Standards of Reporting Trials (CONSORT) guideline for parallel group randomized trials.

### Participants

Recruitment methods included referrals from outpatient clinics at GLA, letter-based recruitment to veterans registered at GLA who met basic selection criteria (eg, age, pain diagnosis) based on a search of data from the VA Corporate Warehouse, and self-referral from posted flyers. Inclusion criteria were veteran status, age 60 to 95 years, and at least 3 months of musculoskeletal pain, including back, leg, or pelvic pain; neck pain or whiplash; temporomandibular joint disorder; fibromyalgia; tension headache; or any combination of these. Exclusion criteria were pain secondary to primary disorders, including cancer, autoimmune disease, sickle cell disease, neuralgia, burn, infection, cauda equina syndrome, gout, migraine or cluster headache, radiography-confirmed hip or knee osteoarthritis, radiculopathy, or electromyography-confirmed carpal or tarsal tunnel syndrome. Also excluded were individuals with psychotic disorder or severe mood disorder not controlled with medications; severe alcohol or substance use disorder; Mini-Mental State Examination (MMSE)^42^ score of 24 or lower (MMSE score ranges from 0 to 30, with higher numbers indicating better cognition); current pain-related litigation; currently receiving EAET or CBT as part of clinical care; participation in CBT in the past 3 months; not fluent in English; and a plan to move from the area in the next 6 months.

Race and ethnicity data were collected by multiple choice self-report using categories in accordance with the National Institutes of Health guidelines. These categories were American Indian or Alaska Native, Asian, Black or African American, Hispanic or Latino, Native Hawaiian or Other Pacific Islander, and White, as well as multiracial or unknown.

### Randomization

An independent team member with no patient contact (M.A.L.) generated the 1:1 computer randomization sequence in blocks of 16 at the start of the trial. The principal investigator (B.C.Y.) and study coordinators (including A.A.) were blinded to the randomization until the next cohort of 16 patients was recruited and ready to be randomized. Participants were randomized to EAET or CBT following the baseline assessment session. Participants learned of their assignment at the first treatment session but remained blinded to study hypotheses for the duration of the trial.

### Interventions

Each treatment included one 90-minute individual session followed by eight 90-minute sessions in small groups with a mean (SD) of 6.3 (1.6) veterans per group. The trial included a total of 10 EAET groups and 10 CBT groups; an EAET group was always conducted concurrently with a CBT group throughout the trial. All sessions took place in person at GLA.

To avoid threats to fidelity, several steps were taken. Both EAET and CBT were presented as effective for chronic pain and had written manuals^43,44^ with similar amounts of discussion, experiential exercises, and homework. Therapists were nested within treatment condition, presenting only the treatment (EAET or CBT) in which they had training and competence. One primary therapist led a complete course of treatment, including individual and group sessions, under the direction of an expert supervisor who monitored treatment adherence and fidelity. Primary therapists for EAET included 1 licensed psychologist, 2 licensed psychiatrists, and 1 psychiatry resident, who became a geriatric psychiatry fellow during the trial. Primary therapists for CBT were 1 licensed psychologist and 3 geriatric psychology postdoctoral residents. Sessions were video recorded, and 25% were rated by an independent fidelity monitor using a standard rating form^39,40^ of topics and activities from EAET and CBT; treatment fidelity was generally rated as high (eTable 1 in Supplement 2).

The EAET followed a manual for the individual session that was developed and piloted for this population^41^ and the group manual developed by Lumley and Schubiner,^43^ which was previously adapted for the population.^41^ The EAET participants were presented with the EAET conceptual model that failure to adaptively process and resolve emotional symptoms—resulting from adverse childhood experiences, trauma, or victimization—can change brain pathways involved in the processing of both pain and emotion, leading to or exacerbating pain symptoms.^19,20,21,22,23,24,25,26,27,28,29,30^ The goals of EAET are to learn that the brain’s perception of pain is strongly influenced by evading trauma- or stress-related emotions (eg, emotional pain or grief, fear, rage, and guilt) and to face and resolve such emotions to reduce or eliminate chronic pain.^26,45^ The individual session presented the therapy model and invited participants to engage in a trial of emotional processing, consisting of experiencing trauma- or stress-related emotions in the body, expressing them in words, and releasing or letting them go in adaptive ways (eg, imaginarily buried or cast out to sea). Group sessions included psychoeducation on pain and emotions, further emotional processing, and social disclosure and validation of emotional experiences. Written homework included identifying stress-symptom connections, expressive writing, and practicing adaptive communication of emotions.

The CBT followed the VA *Cognitive Behavioral Therapy for Chronic Pain* manual,^44^ which was presented with minimal adaptations, although the timing of certain sections had been adjusted by the trial’s CBT supervisor during piloting^41^ to match EAET’s 1 individual and 8 group sessions. The CBT participants were presented with the CBT model that chronic pain often leads to patterns of negative thoughts, feelings, and behaviors that can subsequently worsen pain and functioning. The goal of CBT is to target maladaptive patterns of thinking and behavior by learning alternative, more adaptive pain coping skills. The individual session included a pain and health history and introduction of CBT skills training. Group sessions included psychoeducation on 1 or more pain coping skills (eg, progressive muscle relaxation, guided imagery), experiential practice of a skill, and homework applying skills to daily life and recording progress on worksheets.

### Outcomes

Participants completed self-report paper and pencil outcome measures at baseline, posttreatment (week 10), and the 6-month-follow-up. The primary outcome was change in Brief Pain Inventory pain severity,^46^ which calculates the mean of the worst, least, and average pain in the last week and current pain on scales ranging from 0 to 10. Pain severity response at 3 levels ^31,40,41,47^ was also recorded for pain reduction from baseline: at least 30% (clinically significant), at least 50%, and greater than 70%. Secondary or exploratory outcome measures included PROMIS Anxiety,^48^ Depression,^48^ Fatigue,^49^ General Life Satisfaction (NIH Toolbox),^50^ Pain Interference,^51^ and Sleep Disturbance^52^ Short Forms; the Patient Global Impression of Change (PGIC) Scale^53^ score (at posttreatment and 6-month follow-up); and the Satisfaction with Therapy and Therapist Scale-Revised^54^ (at posttreatment only). In addition, the PTSD Checklist for *Diagnostic and Statistical Manual of Mental Disorders* (Fifth Edition)^55^ was added at the beginning of group 3 at the request of the Data Monitoring Committee.

### Sample Size Calculation

We used an empirical power simulation to estimate the power to detect between-group differences in pain severity change from baseline to posttreatment. Using multivariate normal distributions, we assumed a mean difference of −0.70 points between EAET and CBT, within-time-period SD of 1.6, and correlation of 0.6 (ρ) between baseline and posttreatment. Estimates were based on preliminary data in the population, which demonstrated a mean difference of −0.80 points. Based on these parameters, we assessed for differences using a mixed-effects linear regression model and a 2-sided α level of .05. A sample size of 120 participants (n = 60 per treatment) was needed to demonstrate 80% power to detect a between-group difference in pain severity change over time of −0.70 points. We planned to obtain informed consent from 160 participants to account for an estimated 25% attrition prior to randomization. The actual number of participants who provided written consent was 175 because an additional 15 participants discontinued prior to baseline during the COVID-19 administrative hold.

### Statistical Analysis

All analyses were for the intention-to-treat sample and included all randomized participants. For pain severity and secondary (exploratory) outcomes of anxiety, depression, fatigue, general life satisfaction, pain interference, sleep disturbance, PTSD symptoms, and PGIC score, mixed-effects linear regression models were used with group number (1-10) random intercept, nested patient random intercept with nested time random slope, and clustered robust SEs for group provision of treatment (to account for heteroscedasticity). Fixed effects were treatment (EAET vs CBT), time (modeled as a categorical factor referent to baseline), and time by treatment interaction. For posttreatment satisfaction, mixed-effects linear regressions were used with group number random intercept and clustered robust SEs for group treatment. Fixed effects were treatment (EAET vs CBT).

In prespecified secondary analyses of the primary outcome of pain severity, the percentages of participants achieving 3 pain reduction response levels (at least 30%, at least 50%, and greater than 70% reduction from baseline) were analyzed using mixed-effects logistic regression with group number random intercept and nested patient random intercept. Fixed effects were treatment, time (modeled as a categorical factor referent to baseline), and time by treatment interaction. Although our primary interest was between-condition differences in response within each time point after baseline, data were modeled longitudinally to minimize bias from attrition at the 6-month follow-up. Marginal estimates were used to compute within–time point, between-condition comparisons.

To explore moderation of treatment effect by baseline depression and anxiety, mixed-effects models incorporated additional fixed effects for depression or anxiety into a 3-way interaction of treatment by time by baseline mood. Baseline depression and anxiety were examined as binary variables representing scores above or below the median and as continuous variables. In addition, post hoc analyses explored moderation of treatment effect by PTSD symptoms in the subsample for which the measure was available (groups 3-10).

All statistical analyses were conducted with Stata MP, version 18.0 (StataCorp LLC). A 2-sided value of *P* < .05 was considered statistically significant.

## Results

### Participant Characteristics

**Figure 1.**
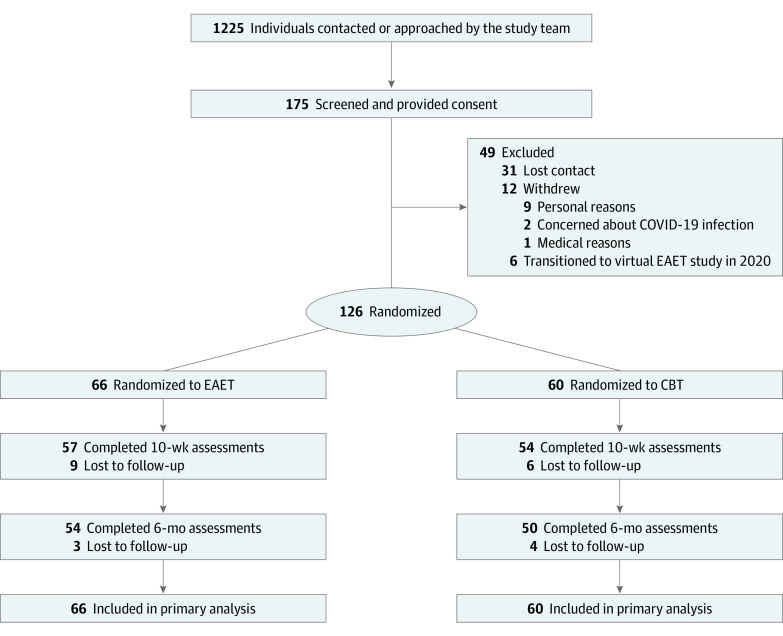
Flow of Participants through the Clinical Trial CBT indicates cognitive behavioral therapy; EAET, emotional awareness and expression therapy.

In total, 1225 individuals were contacted by the study team; 175 completed screening and signed written informed consent (Figure 1). Of these, 31 could not be contacted to be randomized, 12 withdrew for personal or medical reasons, and 6 were transitioned to a virtual EAET pilot study^39^ after the present trial was placed on a COVID-19–related administrative hold. Thus, 126 individuals were randomized, 111 individuals (88%) completed posttreatment assessments, and 104 individuals (82%) completed 6-month follow-up assessments. There were no significant baseline differences between individuals with or without posttreatment or follow-up data (eTable 2 in Supplement 2). All randomized participants attended an individual session and a mean (SD) of 5.5 (2.7) of 8 group sessions.

The full randomized sample had a mean (SD) age of 71.9 (5.9) years, and 116 (92%) were male, and 10 (8%) were female (Table 1). Most participants, 69 (55%), were Black or African American (not of Hispanic origin), 39 (31%) were White (not of Hispanic origin), and 17 (14%) reported being of more than 1 race or ethnicity or their race unknown. In addition, 8 participants (6%) reported Hispanic or Latino ethnicity. The results in the categories American Indian or Alaska Native and Asian or Native Hawaiian or Other Pacific Islander are not reported owing to small numbers. Nearly all participants (121 [96%]) reported back pain, although multiple pain locations were typical; mean (SD) pain duration was 23.3 (17.7) years; and 14 participants (11%) were prescribed opioids. Over two-thirds of participants (87 [69%]) had psychiatric diagnoses, and 47 participants (37%) had PTSD. Participants in the sample had a mean (SD) of 5.0 (2.4) chronic medical conditions, 9.6 (4.8) prescription medications, and MMSE score of 28.6 (1.4).

**Table 1.** Baseline Demographic and Clinical Characteristics and Outcome Measures of All Randomized Participants by Treatment Condition

Characteristic	Participants, No. (%)	
	EAET (n = 66)	CBT (n = 60)
Demographic characteristics		
Age, mean (SD), y	72.6 (5.2)	71.3 (6.6)
Sex		
Female	7 (11)	3 (5)
Male	59 (89)	57 (95)
Race^a^		
American Indian or Alaska Native	NR	NR
Asian or Native Hawaiian or Other Pacific Islander	NR	NR
Black or African American (not of Hispanic origin)	32 (48)	37 (62)
Multiracial or unknown	11 (17)	6 (10)
White (not of Hispanic origin)	22 (33)	17 (28)
Hispanic or Latino ethnicity	6 (9)	2 (3)
Marital status		
Married or partnered	23 (35)	19 (32)
Divorced or separated	25 (38)	25 (42)
Never married or other	18 (27)	16 (27)
Educational level		
≤High school graduate	10 (15)	14 (23)
Some college	36 (55)	26 (43)
≥College graduate	20 (30)	20 (33)
Pain-related characteristics		
Pain location		
Back	63 (95)	58 (97)
Neck	45 (68)	32 (53)
Leg	52 (79)	50 (83)
Pelvic or groin	28 (42)	20 (33)
Temporomandibular joint disorder	11 (17)	10 (17)
Fibromyalgia	3 (5)	2 (3)
Tension headache	19 (29)	15 (25)
Pain duration, mean (SD), y	22.5 (18.2)	24.2 (17.3)
Prescribed opioids at baseline	8 (12)	6 (10)
Other clinical characteristics		
Any psychiatric diagnosis	44 (67)	43 (72)
VA service–connected for PTSD	25 (38)	22 (37)
Nonpain chronic medical conditions, mean (SD), No.	5.0 (2.2)	4.9 (2.7)
Prescription medications, mean (SD), No.	9.9 (5.2)	9.4 (4.3)
Mini-Mental State Examination, mean (SD), score^b^	28.9 (1.1)	28.4 (1.6)
Baseline outcome measure scores, mean (SD)		
BPI pain severity, mean (SD), score^c^	5.97 (1.98)	6.23 (1.63)
PROMIS Anxiety Short Form 7a, mean (SD), score^d^	19.0 (7.0)	19.7 (7.3)
PROMIS Depression Short Form 8a, mean (SD), score^e^	20.6 (7.7)	20.3 (8.5)
PROMIS Fatigue Short Form 7a, mean (SD), score^f^	20.8 (5.4)	21.4 (5.4)
NIH Toolbox General Life Satisfaction Fixed Form B, mean (SD), score^g^	15.1 (4.4)	14.6 (4.1)
PROMIS Pain Interference Short Form 8a, mean (SD), score^h^	27.7 (8.1)	29.6 (7.0)
PROMIS Sleep Disturbance Short Form 8a, mean (SD), score^i^	24.6 (8.2)	24.0 (8.2)
PCL-5, mean (SD), score^j^	28.9 (19.1)	25.5 (18.6)

Abbreviations: BPI, Brief Pain Inventory; CBT, cognitive-behavioral therapy; EAET, emotional awareness and expression therapy; NR, not reported; PCL-5, PTSD Checklist for *DSM-5*; PROMIS, Patient Reported Outcomes Institute Measurement System; PTSD, posttraumatic stress disorder; VA, Veterans Affairs.

^a^ Race and ethnicity data from categories with only a few people were not reported to protect the individuals’ identity.

^b^ Score ranged from 0 to 30, with higher scores indicating better cognition.

^c^ The primary outcome was the average of the 4 pain severity items of the BPI,^46^ which measures current pain and worst, least, and average pain over the last 7 days, each on a scale of 0 to 10, with higher values indicating worse pain.

^d^ The PROMIS Anxiety Short Form 7a^48^ includes 7 items assessing anxiety symptoms over the last 7 days, each rated from 1 to 5 for total scores ranging from 7 to 35; higher scores indicate greater anxiety.

^e^ The PROMIS Depression Short Form^48^ includes 8 items assessing depressive symptoms over the last 7 days, each rated from 1 to 5 for total scores ranging from 8 to 40; higher scores indicate greater depression.

^f^ The PROMIS Fatigue Short Form 7a^49^ includes 7 items assessing symptoms of fatigue over the last 7 days, each rated from 1 to 5 for total scores ranging from 7 to 35; higher scores indicate greater fatigue.

^g^ The NIH Toolbox General Life Satisfaction Fixed Form B^50^ includes 5 items assessing the degree to which participants agree or disagree with statements about life satisfaction; each item is scored from 1 to 5 for total scores between 5 and 25, and higher scores indicate greater life satisfaction.

^h^ The PROMIS Pain Interference Short Form 8a^51^ includes 8 items assessing the interference of pain with mood and activities over the last 7 days, each rated from 1 to 5 for total scores ranging from 8 to 40; higher scores indicate greater pain interference.

^i^ The PROMIS Sleep Disturbance Short Form 8a^52^ includes 8 items assessing sleep disturbance over the last 7 days, each rated from 1 to 5 for total scores ranging from 8 to 40; higher scores indicate greater sleep disturbance.

^j^ The PCL-5^55^ includes 20 items on PTSD symptoms related to the most stressful experience in life, each rated from 0 to 4 for total scores between 0 and 80, with higher scores indicating greater stress. Sample sizes for the PCL-5 are EAET, n = 52; CBT, n = 47.

### Outcomes

**Figure 2.**
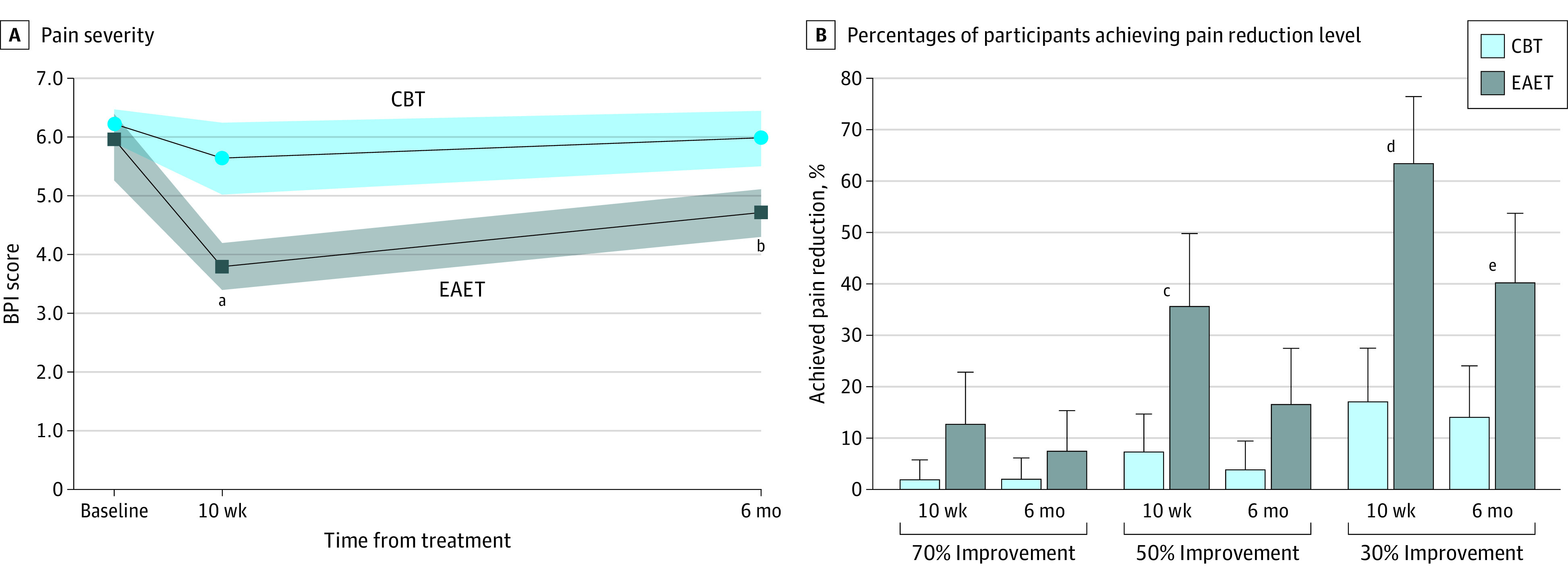
Pain Severity Outcomes A, Shading represents SEs. B, Vertical lines represent upper bound of the 95% CI. CBT indicates cognitive behavioral therapy; EAET, emotional awareness and expression therapy. ^a^*P* < .001. ^b^*P* = .01. ^c^*P* = .002. ^e^*P* < .001. ^e^*P* = .006.

Descriptive statistics for outcomes appear in eTable 3 in Supplement 2. All results for primary analyses are shown in Table 2. For the primary outcome of pain severity (Figure 2A), both treatment conditions showed significant within-condition reductions at posttreatment, with EAET demonstrating a greater reduction in pain severity than CBT (estimate, −1.59 [95% CI, −2.35 to −0.83]; *P* < .001). At the 6-month follow-up, only EAET showed a significant within-condition reduction from baseline, with a difference in the change from baseline favoring EAET over CBT (estimate, −1.01 [95% CI, −1.78 to −0.24]; *P* = .01).

**Table 2.** Primary Analyses Using Mixed-Effects Linear and Logistic Regression Models With Group by Time Interaction

Outcome	Estimate (95% CI)		EAET vs CBT comparison	
	EAET	CBT	Difference or OR, estimate (95% CI)	*P* value
**Primary outcome**				
BPI pain severity				
Change from baseline to posttreatment	−2.18 (−3.00 to −1.37)^a^	−0.60 (−1.13 to −0.06)^b^	−1.59 (−2.35 to −0.83)	<.001
Change from baseline at 6-mo follow-up	−1.26 (−1.83 to −0.68)^a^	−0.25 (−0.72 to 0.23)	−1.01 (−1.78 to −0.24)	.01
At least 30% pain reduction				
Posttreatment, %	63.5 (50.6 to 76.4)	17.1 (6.8 to 27.5)	21.54 (4.66 to 99.56)^c^	<.001
6-mo Follow-up, %	40.3 (26.8 to 53.8)	14.2 (4.3 to 24.0)	7.24 (1.74 to 30.06)^c^	.006
At least 50% pain reduction				
Posttreatment, %	35.7 (21.5 to 49.8)	7.4 (0.0 to 14.7)	11.77 (2.38 to 58.25)^c^	.002
6-mo Follow-up, %	16.6 (5.8 to 27.4)	3.9 (0.0 to 9.4)	6.58 (0.99 to 43.67)^c^	.05
At least 70% pain reduction				
Posttreatment, %	12.8 (2.7 to 22.8)	1.9 (0.0 to 5.8)	13.93 (0.86 to 225.44)^c^	.06
6-mo Follow-up, %	7.5 (0.0 to 15.3)	2.1 (0.0 to 6.1)	5.61 (0.35 to 89.30)^c^	.22
**Secondary outcomes**				
PROMIS Anxiety Short Form 7a				
Change from baseline to posttreatment	−3.13 (−4.56 to −1.70)^a^	−0.64 (−2.18 to 0.89)	−2.49 (−4.30 to −0.68)	.006
Change from baseline at 6-mo follow-up	−3.17 (−4.58 to −1.76)^a^	−1.22 (−2.53 to 0.09)	−1.95 (−4.34 to 0.45)	.11
PROMIS Depression Short Form 8a				
Change from baseline to posttreatment	−5.12 (−6.62 to −3.62)^a^	−2.06 (−4.31 to 0.20)	−3.06 (−5.88 to −0.25)	.03
Change from baseline at 6-mo follow-up	−3.48 (−4.94 to −2.03)^a^	−1.09 (−2.97 to 0.78)	−2.39 (−4.33 to −0.45)	.02
PROMIS Fatigue Short Form 7a				
Change from baseline to posttreatment	−1.97 (−3.00 to −0.93)^a^	−1.19 (−2.44 to 0.06)	−0.78 (−2.33 to 0.78)	.33
Change from baseline at 6-mo follow-up	−1.47 (−2.53 to −0.40)^d^	−1.17 (−2.49 to 0.15)	−0.30 (−1.74 to 1.14)	.69
NIH Toolbox General Life Satisfaction Fixed Form B				
Change from baseline to posttreatment	1.68 (1.02 to 2.34)^a^	0.45 (−0.15 to 1.05)	1.23 (0.36 to 2.10)	.005
Change from baseline at 6-mo follow-up	1.15 (0.82 to 1.48)^a^	0.21 (−0.94 to 1.35)	0.95 (−0.40 to 2.29)	.17
PROMIS Pain Interference Short Form 8a				
Change from baseline to posttreatment	−5.41 (−7.33 to −3.48)^a^	−3.99 (−6.41 to −1.56)^a^	−1.42 (−3.30 to 0.46)	.14
Change from baseline at 6-mo follow-up	−2.87 (−4.96 to −0.78)^d^	−3.08 (−4.59 to −1.57)^a^	0.21 (−1.99 to 2.42)	.85
PROMIS Sleep Disturbance Short Form 8a				
Change from baseline to posttreatment	−2.69 (−4.39 to −0.98)^d^	−0.84 (−2.61 to 0.94)	−1.85 (−4.14 to 0.44)	.11
Change from baseline at 6-mo follow-up	−2.73 (−4.21 to −1.24)^a^	−0.16 (−2.21 to 1.89)	−2.57 (−5.73 to 0.60)	.11
Patient Global Impression of Change^e^				
Posttreatment score	4.67 (4.17 to 5.16)	3.20 (2.79 to 3.61)	1.46 (0.77 to 2.15)	<.001
6-mo Follow-up score	4.01 (3.61 to 4.41)	2.77 (2.34 to 3.21)	1.24 (0.62 to 1.86)	<.001
PCL-5^f^				
Change from baseline to posttreatment	−4.45 (−7.55 to −1.34)^d^	−0.06 (−3.55 to 3.44)	−4.39 (−8.44 to −0.34)	.03
Change from baseline at 6-mo follow-up	−4.07 (−6.26 to −1.88)^a^	−0.31 (−5.28 to 4.66)	−3.76 (−7.83 to 0.32)	.07
Satisfaction with Therapy^g^				
Posttreatment score	24.68 (23.33 to 26.02)	23.59 (22.48 to 24.70)	1.09 (−0.08 to 2.26)	.07
Satisfaction with Therapist^g^				
Posttreatment score	25.73 (24.55 to 26.92)	25.54 (24.60 to 26.48)	0.20 (−0.80 to 1.19)	.70
Global Satisfaction Score^h^				
Posttreatment score	4.30 (4.05 to 4.54)	4.01 (3.83 to 4.19)	0.28 (0.12 to 0.45)	<.001

Abbreviations: BPI, Brief Pain Inventory; CBT, cognitive-behavioral therapy; EAET, emotional awareness and expression therapy; OR, odds ratio; PCL-5, PTSD Checklist for *DSM-5*; PROMIS, Patient Reported Outcomes Institute Measurement System; STTS-R, Satisfaction with Therapy and Therapist Scale-Revised.

^a^ Within condition *P* < .001.

^b^ Within condition *P* < .05.

^c^ Values are ORs (95% CIs).

^d^ Within condition *P* < .01.

^e^ Single item rated from 1 to 7 assessing change in activity limitations, symptoms, emotions, and quality of life since beginning treatment; higher scores indicate greater improvement.

^f^ Sample sizes for the PCL-5 are EAET, n = 52; CBT, n = 47.

^g^ Subscale of the STTS-R^54^ includes 6 items rated from 1 to 5 for possible scores ranging from 6 to 30; higher scores indicate better satisfaction.

^h^ Single item of the STTS-R^54^ rated from 1 to 5; higher scores indicate better satisfaction.

At posttreatment, a significantly greater percentage of EAET vs CBT participants had at least 30% pain reduction (63% vs 17%; odds ratio [OR], 21.54 [95% CI, 4.66-99.56]; *P* < .001) and at least 50% pain reduction (35% vs 7%; OR, 11.77 [95% CI, 2.38-58.25]; *P* = .002). At the 6-month follow-up, a greater percentage of EAET vs CBT participants maintained at least 30% pain reduction (41% vs 14%; OR, 7.24 [95% CI, 1.74-30.06]; *P* = .006; Figure 2B).

For secondary or exploratory outcomes, significant between-group differences favoring EAET over CBT in baseline to posttreatment change were observed for anxiety (estimate, −2.49 [95% CI, −4.30 to −0.68]; *P* = .006), depression (estimate, −3.06 [95% CI, −5.88 to −0.25]; *P* = .03), general life satisfaction (estimate, 1.23 [95% CI, 0.36-2.10]; *P* = .005), PTSD symptoms (estimate, −4.39 [95% CI, −8.44 to −0.34]; *P* = .03), PGIC score (estimate, 1.46 [95% CI, 0.77-2.15]; *P* < .001), and global satisfaction (estimate, 0.28 [95% CI, 0.12-0.45]; *P* < .001). Significant differences favoring EAET over CBT were observed in baseline to 6-month follow-up change for depression (estimate, −2.39 [95% CI, −4.33 to −0.45]; *P* = .02) and PGIC score (estimate, 1.24 [95% CI, 0.62-1.86]; *P* < .001). No other reliable differences were observed.

### Moderation

**Figure 3.**
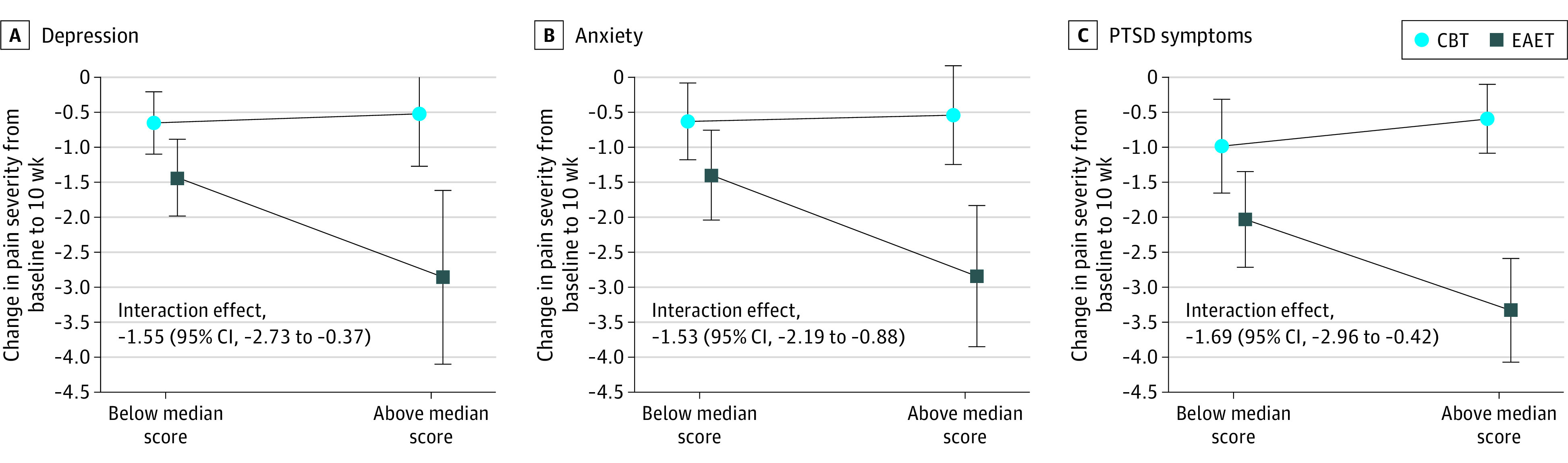
Moderation of Pain Severity Reduction between Treatment Conditions by Baseline Depression, Anxiety, and PTSD Symptom Scores The interaction effect estimate is the difference in the differences-in-difference estimates between the 2 treatment conditions. CBT indicates cognitive-behavioral therapy; EAET, emotional awareness and expression therapy; PTSD, posttraumatic stress disorder.

When examining above-median vs below-median baseline depression, the differential depression effect for EAET (difference-in-differences [DID] estimate, –1.42) was different for CBT (DID, 0.13) (difference in DID estimate for the interaction effect, −1.55 [95% CI, −2.73 to −0.37]; *P* = .01), suggesting a greater effect of baseline depression on reduction in pain severity for EAET relative to CBT (Figure 3A). When using baseline depression as a continuous moderator, for a 1-SD increase in baseline depression, the between-condition reduction in pain severity at posttreatment was greater for EAET than CBT (estimate, −0.83 [95% CI, −1.32 to −0.35]; *P* < .001). Similar effects were observed when examining above-median vs below-median anxiety (difference in DID estimate, −1.53 [95% CI, −2.19 to −0.88]; *P* < .001; Figure 3B) and PTSD (difference in DID estimate, −1.69 [95% CI, −2.96 to −0.42]; *P* = .009; Figure 3C). See eTable 4 in Supplement 2 for further detail.

## Discussion

In this randomized clinical trial among a racially and ethnically diverse cohort of veterans 60 to 95 years of age with chronic musculoskeletal pain, EAET was superior to CBT on the primary outcome of reduction in pain severity from baseline to posttreatment and 6-month follow-up. Compared with CBT, EAET led to a significantly greater percentage of participants who reached the benchmarks of at least 30% and 50% pain reduction at posttreatment and at least 30% pain reduction at follow-up. At posttreatment, EAET also demonstrated greater improvements than CBT on most secondary or exploratory outcomes—anxiety, depression, life satisfaction, PTSD symptoms, patient global impression of change, and global satisfaction—and maintained advantages on depression and global impression 6 months posttreatment. Moderation analyses revealed that patients with higher baseline symptoms of depression, anxiety, or PTSD had particularly robust pain reduction following EAET but not CBT.

This trial is the first full-scale evaluation of EAET, to our knowledge, in a medically or psychiatrically complex, racially and ethnically diverse, older sample comprising predominantly men. Findings align with prior smaller controlled or uncontrolled trials of EAET that have shown large effect size reductions in pain severity^39,41^ and high response rates, with up to two-thirds of patients achieving at least a 30% pain reduction.^31,39,47^ The present trial is only the third to directly compare EAET with CBT. A small trial in the same population^41^ showed superiority of EAET to CBT on pain severity and anxiety, and a large trial of patients with fibromyalgia^40^ showed that EAET was superior to CBT on several secondary outcomes (fibromyalgia symptoms, widespread pain, and 50% pain reduction at 6-month follow-up), but not on mean pain severity. Moreover, while previous research indicated a less favorable response to CBT among patients with the common comorbidities of depression and anxiety,^18^ the current trial indicated that EAET is especially effective for such patients. Thus, the current trial provides the strongest evidence to date supporting the superiority of EAET over CBT.

The results of this trial lend credence to 2 interrelated conceptual models of chronic pain proposing that the condition may be substantially reduced and, in some cases, eliminated by helping patients (1) change beliefs about the potential amelioration of pain via psychological techniques,^56^ and (2) process difficult and often trauma-related emotions in specific ways.^57^ The EAET integrates both models and targets both mechanisms.^26^ The superiority of EAET vs CBT suggests the potential benefits of further incorporating these concepts into mainstream clinical pain medicine.

### Limitations

This study has several limitations. First, the trial focused on older veterans, and findings may not be generalizable to younger patients or to nonveterans; the trial should be replicated in younger adults. Second, the trial included mostly men and a high percentage of patients with PTSD, which may have improved response to EAET. However, the sample size precluded sufficient power for a legitimate test of these issues. Third, study therapists’ backgrounds were not exactly matched between the 2 conditions. Fourth, all sessions were conducted in person; thus, whether EAET is similarly effective via telehealth is unknown. Fifth, the trial excluded patients with cognitive impairment, and these patients should be examined in future research.

## Conclusions

This randomized clinical trial found that among a racially and ethnically diverse cohort of older adults with chronic musculoskeletal pain, time-limited, group-based EAET was superior to CBT on posttreatment improvements in pain severity, anxiety, depression, life satisfaction, PTSD symptoms, global satisfaction, and PGIC. Improvements in pain severity, depression, and PGIC were sustained for many participants 6 months following the intervention. In contrast to participants in CBT, participants undergoing EAET who had higher baseline depression, anxiety, and PTSD symptoms had particularly robust posttreatment pain reduction. The results suggest that EAET is a preferred intervention for medically and psychiatrically complex older patients with pain. The societal burden of chronic pain may be eased by further incorporating EAET principles into mainstream clinical pain medicine.

## Notes

Supplement 1.
Trial Protocol

Supplement 2. **eTable 1.** Fidelity ratings for each treatment **eTable 2.** Differences between those with and without missing posttreatment or follow-up **eTable 3.** Descriptive statistics for primary and secondary outcomes **eTable 4.** Full results from moderation analyses on change in pain severity

Supplement 3.
Data Sharing Statement
