# Supplementary material for: Emotional Awareness and Expression Therapy vs Cognitive Behavioral Therapy for Chronic Pain in Older Veterans: A Randomized Clinical Trial
Source: JAMA Netw Open. 2024 Jun 13;7(6):e2415842. doi: 10.1001/jamanetworkopen.2024.15842 (PMC11177167; doi:10.1001/jamanetworkopen.2024.15842)
Supplement: Supplement 1. — Trial Protocol [file jamanetwopen-e2415842-s001.pdf]

# Optimizing Psychotherapy for Older Veterans with Chronic Pain

**Funding Agency**: CSR&D Career Development Award

**Principal Investigator**: Brandon C. Yarns, MD, MS

**Version 5**: February 28, 2020

**Abstract:**

The overarching goal of the proposed research is to learn how to optimize psychotherapy for those Veterans most in need and most likely to benefit from psychotherapy, older Veterans with chronic musculoskeletal pain. Chronic pain is a critical healthcare challenge, as the condition affects 50% of all Veterans and affects older Veterans most commonly, severely, and persistently. For years, chronic pain treatment has been notoriously difficult at VA and elsewhere, especially in light of the recent “opioid crisis,” in which opioid analgesics, previously a mainstay of chronic pain treatment, have come under increased scrutiny. In response, CDC, VA/DoD, and some experts have called for enhancing and expanding psychosocial treatment options for chronic pain, such as psychotherapy, which are low risk for older Veterans who frequently have multiple medical comorbidities and are taking multiple medications.

Yet standard VA psychotherapy approaches, such as Cognitive Behavior Therapy (CBT), have shown modest benefits for Veterans on pain and other related outcomes, such as mood, anxiety, and sleep. In contrast, a novel psychotherapy approach, Emotional Awareness and Expression Therapy (EAET), has shown medium to large benefits for some chronic pain patients. Whereas CBT improves pain and negative emotion by teaching patients cognitive and behavioral coping skills, affecting brain regions that enhance “cognitive control” of pain, EAET operates primarily through emotion regulation, which is thought to influence brain regions and circuits that modulate both physical pain and emotion—a mechanism absent from existing approaches. The literature and our pilot data indicate that patients who express emotional distress at baseline, such as high anxiety and depressive symptoms, may be particularly likely to benefit from EAET’s emotion regulation approach, whereas patients who express less emotional distress may derive more benefit from an approach like CBT, which does not require ready access to emotions.

The proposed randomized clinical trial tests the hypothesis that EAET is superior to CBT on reduction in mean pain severity and other outcomes derived from IMMPACT. To examine which patients are most likely to benefit, this research also tests whether greater baseline emotional distress (using measures of anxiety and depression) predicts stronger benefits from EAET and whether lower baseline emotional distress predicts stronger benefits from CBT. Finally, this research explores whether the benefits of EAET are mediated by improved emotion regulation the benefits of CBT are mediated by improved cognitive and behavioral coping, and whether the benefits of both are mediated by a stronger working alliance. We plan to enroll 160 multi-ethnic/multi-racial older Veterans (age 60-95 years) with chronic musculoskeletal pain at the West Los Angeles VA Medical Center.

This research can introduce an additional, potentially more effective format of psychotherapy at VA so that more Veterans with chronic pain can respond. In addition, this research can lead to better treatment targeting and enhance our understanding of how psychotherapy treatments work. Finally, this research can facilitate the development of critical skills for the PI in psychotherapy research and pain management and enhance his ability to effect positive change for older Veterans.

**List of Abbreviations:**

AAGP – American Association for Geriatric Psychiatry
APA – American Psychoanalytic Association
APS – American Psychosomatic Society
ACOS/R – Associate Chief of Staff for Research
ACT – Acceptance and Commitment Therapy
BPI – Brief Pain Inventory
CBT – Cognitive Behavior Therapy
CBT-CP – Cognitive Behavior Therapy for Chronic Pain
CDA – Career Development Award
CDC – Centers for Disease Control and Prevention
CSR&D – Clinical Science Research and Development
DoD – Department of Defense
EAET – Emotional Awareness and Expression Therapy
EBP – Evidence-Based Psychotherapy
ECT – Electroconvulsive Therapy
EMG – Electromyography
fMRI – Functional Magnetic Resonance Imaging
GLMM – General Linear Mixed Model
GPOP – Geriatric Psychiatry Outpatient Program
HIPAA – Health Insurance Portability and Accountability Act
IBM – International Business Machines Corporation
IMMPACT – Initiative on Methods, Measurement, and Pain Assessment in Clinical Trials
IEDTA – International Experiential Dynamic Therapy Association
IPA – International Psychoanalytical Association
IRB – Institutional Review Board
ISTDP – Intensive Short-Term Dynamic Psychotherapy
ITT – Intent to Treat
LGBT – Lesbian, Gay, Bisexual, and Transgender
MAR – Missing at Random
MCAR – Missing Completely at Random
MMSE – Mini-Mental State Examination
MoCA – Montreal Cognitive Assessment
NIH – National Institutes of Health
OBSSR – Office of Behavioral and Social Sciences Research
OPTIMUM – Optimizing Outcomes of Treatment-Resistant Depression in Older Adults
PAST-FM – Pain and Stress Treatment for Fibromyalgia
PCO – Patient-Centered Outcomes
PCORI – Patient Centered Outcomes Research Institute
PGY – Post Graduate Year
PI – Principal Investigator
PROMIS – Patient Reported Outcomes Measurement Information System
PSS – Post-Traumatic Stress Disorder Symptom Scale
PTSD – Post-Traumatic Stress Disorder
RCT – Randomized Clinical Trial
SBCC – Statistical/Biomathematical Consulting Clinic
SD – Standard Deviation
SO – Sheryl Osato, PhD (Lead CBT Interventionist)
SPSS – Statistical Package for the Social Sciences
STTS-R – Satisfaction with Therapy and Therapist Scale—Revised

UCLA – University of California, Los Angeles
UNM – University of New Mexico
VA – Veterans Affairs
VAGLAHS – VA Greater Los Angeles Healthcare System
WLA – West Los Angeles VA Medical Center

## Contents

Protocol Title: Optimizing Psychotherapy for Older Veterans with Chronic Pain ........ 6
1.0 Study Personnel ........ 6
2.0 Introduction ........ 6
3.0 Objectives ........ 10
4.0 Resources and Personnel ........ 11
5.0 Study Procedures ........ 11
5.1 Study Design ........ 11
5.2 Recruitment Methods ........ 12
5.3 Informed Consent Procedures ........ 13
5.4 Inclusion/Exclusion Criteria ........ 13
5.5 Study Evaluations ........ 14
5.6 Interventions and Interventionists ........ 16
5.7 Data Analysis ........ 19
5.8 Withdrawal of Subjects ........ 21
6.0 Reporting/Risks/Safety ........ 22
7.0 Privacy and Confidentiality ........ 23
8.0 Communication Plan ........ 24
9.0 References ........ 26

**Protocol Title: Optimizing Psychotherapy for Older Veterans with Chronic Pain**

## 1.0 Study Personnel

**Principal Investigator:** Brandon C. Yarns, MD, MS; Staff Psychiatrist, West Los Angeles VA Medical Center, 11301 Wilshire Blvd., Bldg. 401, Rm. A236; Ph: 424-279-8439; Email: brandon.yarns@va.gov

**Co-Investigator:** David L. Sultzer, MD; Staff Psychiatrist, West Los Angeles VA Medical Center, 11301 Wilshire Blvd., Bldg. 158, Rm. 166, Los Angeles, CA, 90073; Ph: 310-268-3708; Email: david.sultzer@va.gov

## 2.0 Introduction

**Scientific Background and Rationale:**

The proposed Career Development Award research is a randomized clinical trial comparing Emotional Awareness and Expression Therapy (EAET) with Cognitive Behavior Therapy (CBT) on pain severity and other outcomes. It also proposes to test differences in emotion regulation as predictors of differential treatment response and explore mediators of response to each treatment. Chronic pain affects as many as 50% of male Veterans and 75% of female Veterans and is one of the costliest disorders treated in Veterans Affairs (VA) settings (1-3). Older male and female Veterans are most commonly and severely affected by chronic pain, with 80% of Vietnam-era Veterans reporting chronic pain (4).

For years, the treatment of chronic pain has been notoriously difficult at VA and elsewhere, with opioid analgesics, previously a mainstay of chronic pain treatment, coming under increasing scrutiny, especially in light of the burgeoning “opioid crisis” (5-7). In fact, opioid use disorders are almost 7-fold higher in the VA population compared to commercial health plans (8), and although younger Veterans have higher rates of opioid use disorders, older adults with these disorders are at significantly higher risk of all-cause mortality (9). Elders who use high-dose opioids also have an annual fracture risk approaching 10% (10).

Scientists and policy leaders advocate psychotherapy as a crucial tool to help combat chronic pain and the opioid crisis at VA (6, 11). In the past year, the CDC, VA, and DoD all recommended psychotherapy as a first-line treatment for chronic pain (11, 12). Cognitive Behavior Therapy (CBT) is the most widespread psychotherapy approach currently in use for chronic pain at VA (6). VA has implemented CBT nationally as part of the VA Evidence-Based Psychotherapy (EBP) Training Program, which trains psychologists, psychiatrists, and other mental health professionals to perform this evidence-based approach (6, 13).

**Current Gaps in Knowledge of How to Treat Chronic Pain with Psychotherapy and a New Way Forward:**

Although a VA study indicated overall statistically significant reductions in pain severity and associated patient-centered outcomes (PCO) using VA's CBT for chronic pain manual, average improvement in 7-day worst pain was only -0.34 points, baseline to post-treatment, on an 11-point (0-10) scale (6). In addition, two meta-analyses investigating CBT for chronic pain found only modest short-term reductions in pain severity, disability, depression, and catastrophic thinking in trials that compared CBT to no treatment, and small positive effects on disability and catastrophizing, but not on pain or mood, when comparing CBT to active controls (14). Among chronic pain patients with the common comorbidities of depression or a trauma history—especially common among Veterans with chronic pain—CBT may be particularly ineffective (15).

Acceptance and mindfulness-based approaches to chronic pain have also been of recent interest at VA (16, 17). These approaches comprise a broad category that includes acceptance and commitment therapy (ACT) and other mindfulness approaches such as mindfulness-based stress reduction (18). VA has implemented ACT for depression as part of the EBP Training Program but not for chronic pain (19). Similar to CBT, acceptance and mindfulness-based approaches are effective beyond no treatment or usual care, and may have positive effects on mood and functioning, but still have little impact on pain severity (17, 20-22).

In contrast, promising research on an innovative psychological treatment, Emotional Awareness and Expression Therapy (EAET), has shown that some chronic pain patients who receive psychotherapy can achieve medium-to-large benefits on pain severity and other outcomes (23-25). EAET is grounded in exposure-based, experiential, and psychodynamic theories and uses innovative emotion-focused techniques to reduce stress and alter central nervous system processes to reduce or eliminate pain (26). A recent large study, PAST-FM, showed significantly greater rates of 50% pain reduction at both post-treatment and 6-month follow-up for EAET compared to either CBT or education control for adult outpatient non-Veterans with fibromyalgia, a disorder of widespread musculoskeletal pain (27). Additionally, in a series of 72 adult patients with chronic musculoskeletal pain conditions who received EAET, two-thirds of patients achieved at least 30% pain reduction and one-third of patients had at least 70% pain reduction—rates of success that are almost never found with CBT (23). Finally, in our pilot project at VA Greater Los Angeles, older Veterans with chronic musculoskeletal pain who received EAET had a reduction in mean pain severity nearly three times greater than those who received CBT: -1.44 versus -0.39.

**Rationale for the Population Chosen:**

In order to begin the process of optimizing the psychosocial treatment of chronic pain at VA, the proposed research uses a sample of Veterans likely to respond to psychotherapy, older Veterans. First, according to theory, developmentally older adults are at a phase of life in which they often desire to reflect and process issues, such as is the case in psychotherapy (31). Second, group cognitive behavioral and psychodynamic psychotherapy treatments have been found to be beneficial in depressed older adult Veterans and depressed caregivers in two separate trials at the Palo Alto VA (32, 33). Finally, psychotherapy is suited to older adult Veterans because older adults have been shown to overwhelmingly prefer psychosocial treatments of any modality to pharmacologic treatments (34, 35).

**Individual Differences in Response to Cognitive/Behavioral and Emotional Interventions and Proposed Moderators:**

Chronic pain is frequently accompanied by many comorbid psychiatric symptoms, including depression and anxiety (36-38). Although individual difference research in chronic pain is in its infancy, it is important—and in keeping with recent calls for "personalized medicine" (e.g., (39))—to investigate what factors predict response to different psychosocial treatments. In the literature, higher baseline depression scores predicted greater pain reduction among adults with chronic musculoskeletal pain who completed EAET (23). In addition, in our pilot work, higher baseline anxiety was also associated with greater pain reduction for EAET patients. One possible explanation for these findings is that patients who report higher anxiety or depression, or patients who express greater emotional distress, may be more able to readily access and work with the affective components of pain, which provides the substrate that is amenable to this emotion-focused therapeutic modality.

In contrast, some data suggest that patients with high emotional distress, using measurements of anxiety and depressive symptoms, may have less likelihood of benefit from CBT for chronic pain (15). Theoretically, this may be explained because patients in less emotional distress may be more amenable to learning cognitive and behavioral coping skills in CBT and less capable of working with a treatment such as EAET, which requires ready access to emotions. Thus, this research will investigate these theoretical and empirical findings in our population by examining whether patients with higher emotional distress at baseline—depression or anxiety—have stronger benefits from EAET on primary and secondary outcomes, and Veterans who express lower baseline emotional distress have stronger benefits from CBT.

The measurement of emotional distress commonly uses assessments of depression and anxiety as proxies for distress, such as in the Patient-Reported Outcomes Measurement Information System (PROMIS), which relied on comprehensive literature searches and qualitative item analysis to define item banks for depression and anxiety to, in part, represent emotional distress (40).

**Two Interventions with Largely Distinct Mechanisms of Action to Influence the Central Nervous System Aspects of Chronic Musculoskeletal Pain:**

Although they may share some similarities, such as the importance of a positive working alliance, CBT and EAET are hypothesized to operate through largely distinct mechanisms of action, which this research aims to explore.

EAET is hypothesized to achieve reduction of pain and related symptoms through the mechanism of improving emotion regulation. Emotion regulation includes at least two processes and is thought to be impaired following stressful or traumatic life experiences (41). The first process, emotional awareness, involves the ability to attend to, differentiate among, and label feelings. An example of impaired emotional awareness is the case of alexithymia, which has been linked to a variety of negative health outcomes, including worse pain (42). More specifically, an inability to differentiate among feelings has been linked to more stress and pain in several small experiments (43). The second component of emotion regulation is healthy emotional expression, which involves an ability to disclose and express one's feelings verbally and behaviorally in adaptive ways. Substantial evidence shows that secrecy and decreased expression of emotions has negative health effects (44-48). For example, elegant studies by Burns demonstrate that impaired healthy expression of anger, in particular, increases subjective and behavioral pain as well as the activity of the relevant muscles (49-51). Emotion regulation is thought

to operate primarily through brain structures involved in processing both the affective component of pain and other emotions. These structures include the insula, dorsal anterior cingulate cortex, thalamus, and secondary somatosensory cortex (52).

Some research suggests that interventions can improve pain through the mechanism of improving emotion regulation (i.e., emotional awareness and expression). There were early reports of substantial success of approaches that encourage pain patients to acknowledge psychological conflicts and emotions (53, 54), but the reliability of these findings is limited by patient selection bias and lack of controls. More recent studies with greater methodological rigor suggest that experiencing rather than avoiding one's negative emotional experience has benefits for pain. One approach, including writing repeatedly about one's private stressful experiences and avoided emotions (i.e. written emotional disclosure) has shown benefits in controlled studies for a range of populations and outcomes (55, 56). Specifically, emotional disclosure has led to improvements in chronic pelvic pain (57), several specific chronic pain conditions (58), and fibromyalgia, a disorder of widespread chronic musculoskeletal pain (59). In addition, there are positive trials of EAET specifically in adults with chronic musculoskeletal pain (23) and fibromyalgia (24). Finally, as described below in the Preliminary Studies section, our pilot data has demonstrated even greater benefits of EAET compared to CBT in older Veterans with chronic musculoskeletal pain. The proposed research tests whether EAET actually works through the hypothesized mechanism of improving emotion regulation, using measures of ambivalence (hypothesized to decrease) and emotional approach coping (hypothesized to increase).

In contrast, CBT for chronic pain is hypothesized to achieve improvement in pain, dysfunction, and distress through the mechanism of enhancing patients' cognitive and behavioral coping (60, 61). Techniques to improve cognitive coping include cognitive control (e.g., distraction), reframing of cognitions, and logical problem solving, whereas techniques to improve behavioral coping include arousal reduction (e.g., relaxation) and increasing positive experiences. The proposed research tests whether CBT actually works through the mechanism of improving cognitive coping skills (using measurement of pain catastrophizing) and behavioral pain coping (using measurement of adaptive coping). Basic science research indicates that CBT primarily acts through improving the volume and functioning of prefrontal brain regions, rather than brain regions that are involved in emotional processing, as in EAET (62, 63).

In spite of these distinct hypothesized mechanisms of action, both CBT and EAET are proposed to share some mechanistic qualities. For instance, both psychodynamic treatments and CBT are hypothesized to operate, in part, through the strength of the working alliance (64, 65). Thus, the proposed research also tests whether response to both treatments is mediated by a positive working alliance.

**Significance of the Proposed Research:**

Given the limited efficacy of available psychosocial treatments for chronic pain and the recent opioid crisis, it is vital that novel approaches be developed and tested. There is substantial evidence that emotional avoidance resulting from failure to process stressful experiences and their accompanying emotions contribute to the pain and symptoms of many individuals with chronic pain, suggesting the need for a treatment such as EAET that largely targets these processes. Thus, the proposed research holds substantial promise to advance clinical practice and treatment outcomes, as well as theory and future research.

As suggested by theory, empirical research, and our pilot data, an EAET approach has great potential to improve the health of older Veterans with chronic musculoskeletal pain, thus filling a gap in current pharmacologic, physical, and cognitive-behavioral approaches. This research can make available an innovative, empirically-supported therapy, including a written protocol, for use in treating these Veterans. Comparing EAET to a standard intervention is key to determining whether the innovative EAET approach is superior to what is currently available, and also whether different subsets of older Veterans, such as those with different levels of emotional distress, respond to these two different interventions. If these aims are supported, then our ability to tailor interventions for specific patients, or match Veterans to optimal treatments, will be greatly enhanced. Furthermore, the strong recruitment of racial/ethnic minorities in our pilot work is a further strength of this research, which will focus on a multi-ethnic/multi-racial Veteran population and the importance of understanding and improving treatment approaches across Veterans of all races and ethnicities.

With respect to theory, this research will advance our understanding of the distinct and shared mechanisms of EAET and CBT and the role played by emotional factors in chronic pain. Indeed, whether stress and emotional avoidance contribute to chronic pain—as suggested by correlational data—needs to be tested experimentally, such as with this clinical trial. This research will examine hypothesized mediators of both EAET and CBT, thus providing an optimal test of whether each intervention operates as theoretically specified, or whether these interventions change more general processes (e.g., working alliance). In addition, this research will enhance our understanding of individual differences in emotional regulation and how such differences map onto emotional and cognitive-behavioral interventions.

This work will also inform and direct future research. For example, a subsequent study during the Merit Review period can focus more on therapist factors by examining whether typical patients will accept and benefit from this intervention when it is presented by therapists with little or no prior experience with emotional interventions. Future research may also focus on how best to implement EAET in VA, whether through the Evidence-Based Psychotherapy Training Program or elsewhere. In addition, future studies can test whether EAET is effective for other populations of Veterans with chronic pain and Veterans with other syndromes involving elevated stress and emotional dysregulation, such as irritable bowel syndrome and chronic fatigue. Future research can also test alternative EAET formats such as self-help, telepsychiatry, and internet-based delivery methods. Given current movements toward integration, ultimately one should develop a unified or integrative protocol that combines cognitive-behavioral and emotional components along with pharmacologic therapies in an effort to have the greatest coverage for the greatest number of patients. Indeed, empirically-based treatment integration has the potential to give new direction to a field that has been marked by isolated theories and narrow treatments, and the proposed research can contribute to that integration.

## 3.0 Objectives

The Specific Aims for this study are:

**Aim 1: Main Effects.** Test the hypothesis that EAET is superior to VA standard treatment (CBT) in 160 older Veterans (age 60-95 years) with chronic musculoskeletal pain on the following PCO:

a. **Primary outcome:** change in mean pain severity baseline to post-treatment.

b. **Secondary outcomes:** change in pain interference, mood, anxiety, PTSD symptoms, meaning/purpose, sleep disturbance, fatigue, patient global impression of change, and patient satisfaction baseline to post-treatment and baseline to 6-month follow-up and change in mean pain severity baseline to 6-month follow-up.

**Aim 2: Moderation of the Interventions.** Test the hypothesis that EAET will have stronger benefits for patients who express more baseline emotional distress (using greater depression and anxiety as proxies for emotional distress), whereas CBT will have stronger benefits for patients who express less baseline emotional distress (using greater depression and anxiety as proxies).

**Aim 3: Mediation of the Interventions.** Explore hypotheses that the benefits of EAET are mediated by improved emotion regulation (as reflected in several measures of emotion regulation), the benefits of CBT are mediated by improved cognitive and behavioral coping (as reflected in several measures of coping), and both interventions are mediated by a positive working alliance.

## 4.0 Resources and Personnel

All study procedures will take place at the West Los Angeles VA Medical Center. The PI will oversee all aspects of the research, including recruitment/screening, informed consent, performance of study interventions, and data collection and analysis. Study coordinators will conduct procedures involving recruitment/screening, informed consent, data collection and analysis. Interventionists will conduct study interventions, as described below in Section 5.6. The Co-Investigator, Dr. Sultzer, will assist the PI in overseeing all aspects of the research.

## 5.0 Study Procedures

### 5.1 Study Design

The project is a randomized superiority trial that compares change in mean pain severity and other outcomes in older Veterans (age 60-95 years) with chronic musculoskeletal pain who receive either Emotional Awareness and Expression Therapy (EAET) or Cognitive Behavior Therapy (CBT). The study includes 160 total participants, 80 randomly assigned to EAET and 80 to CBT. Each participant receives one 90-minute individual session and eight 90-minute group sessions based on the treatment condition (EAET or CBT) to which he/she is randomly assigned. Each EAET group at a time runs concurrently with one CBT group throughout the study. Group sessions consist of small groups of eight Veterans. All participants complete self-report questionnaires on pain, physical and psychiatric symptoms, and emotional well-being at baseline, post-treatment, and 6-month follow-up. Participants also complete self-report questionnaires on emotional approach coping, ambivalence over emotional expression, pain catastrophizing, and adaptive pain coping skills at baseline, post-treatment, and 6-month follow-up. Participants complete a working alliance questionnaire at post-treatment. The data are used to assess the superiority of EAET compared to CBT on change in mean pain severity scores from baseline to post-treatment (primary outcome) and 6-month follow-up and change in other patient-centered outcomes (PCO) from baseline to post-treatment and 6 month-follow-up (secondary outcomes). In addition, the data are used to test mediation of EAET by improved emotion regulation (greater emotional approach coping and decreased ambivalence over

emotional expression), mediation of CBT by improved cognitive and behavioral coping (decreased catastrophizing and increased pain coping skills), and mediation of both treatments by strength of the working alliance. Finally, the data are used to test whether higher baseline depression and anxiety predict response to EAET and lower baseline depression and anxiety predict response to CBT.

### 5.2 Recruitment Methods

**Participants:**

The study includes 160 Veterans age 60-95 years (2 treatment conditions x 10 groups x 8 Veterans = 160) with chronic musculoskeletal pain. Veterans are recruited from outpatient clinical sites at West Los Angeles Veterans Affairs Medical Center (WLA), the largest VA Medical Center on the West Coast. We have already partnered with several clinical sites, including Geriatric Psychiatry, Geriatrics, Chronic Pain, Interventional Pain, Women's, and Primary Care Clinics, for our pilot, and plan to use these sites for this project as well. In our pilot, we were able to recruit 48 participants from our clinical sites in 30 weeks. For the proposed trial we have a similar recruitment schedule of approximately 1-2 Veterans per week, in order to complete all treatments and 6-month follow-ups by the end of Year 4, leaving Year 5 for data clean-up and analysis. The schedule in Table 1 indicates recruitment goals for the duration of the study.

| **Table 1: Enrollment for Each Year** | Year 1 | Year 2 | Year 3 | Year 4 | Year 5 |
|---|---|---|---|---|---|
| Screening, Baseline Assessments, **EAET,** Post-Treatment and 6-month Follow-Up Assessments | 24 | 24 | 16 | 16 | - |
| Screening, Baseline Assessments, **CBT**, Post-Treatment and 6-month Follow-Up Assessments | 24 | 24 | 16 | 16 | - |

**Recruitment and Screening Procedures:**

Approved clinician and patient flyers are distributed to clinic staff in participating programs (see attached). Before clinic, a medical chart review is performed to determine whether potential Veteran participants meet basic inclusion/exclusion criteria. Clinicians not associated with the study will passively recruit patients by handing them a flyer and referring them to the research team. No Veteran is approached by research staff without having previously agreed to such contact with his/her clinician.

In addition, we will screen additional medical charts using a VA Data Warehouse generated list of veterans aged 60 and older, who carry a diagnosis of low back pain, and are actively receiving care from a VA PCP. Patients identified as eligible this way will be mailed a letter and a flyer about the study. Only patients who reach out to the research team will be considered for enrollment in the study and will not be contacted otherwise.

Finally, we post flyers on VA bulletin boards and at identified locations in the aforementioned clinics, and prospective Veterans are self-referred to research staff through these flyers. A waiver for consent and documentation of consent for recruitment/screening only is being sought from the IRB. Patients who are unable to give informed consent or have a legal conservator are not included in the study. Only in cases in which a patient's capacity to provide informed consent is questionable (according to the clinician's best judgement) will they be required to complete the UCLA Capacity for Consent Form to determine whether they may be included in the study. Patients who have assigned payees are compensated accordingly.

**Payments:**

After completing the baseline visit (baseline questionnaires), participants will receive $50. After completing posttreatment questionnaires (Week 9), participants will receive $50. After completing 6-month follow-up questionnaires (Week 39), participants will receive $50. Participants receive a check, which is mailed within three weeks of completing the aforementioned study procedures.

Proposed payments are provided for participant time, inconvenience, and transportation to complete research procedures (i.e., questionnaires). Substantial time is needed in order to complete the battery of questionnaires. In addition, driving in Los Angeles can be challenging, with many participants driving a considerable distance. Thus, payments are modest. Participants are not reimbursed for attendance at psychotherapy sessions.

### 5.3 Informed Consent Procedures

After a Veteran has expressed interest in the study, the PI or other research team member discusses the details of the study, including study procedures, risks, and benefits, with the Veteran by phone or in person. There is an opportunity to ask questions, to have information clarified, and for potential participants to discuss the study with other family members. Standardized demographics and medical history are obtained, as well as psychiatric diagnoses and substance use disorder status. All patients complete the MMSE for screening purposes. If the potential participant meets screening criteria, he/she is invited to enroll as a primary research participant. Those who fail to meet the inclusion/exclusion requirements are thanked for their time. Interested participants come to meet in person with the PI or another research team member to enroll in the study. Consent and HIPAA forms are reviewed with the participant prior to the start of the study. As the need arises, decision-making capacity is assessed by a member of the research team who is a psychiatrist or licensed psychologist. The UCLA Capacity to Consent Form will be used for this purpose. Baseline assessments are then completed. Finally, the Veteran is randomized and scheduled for an individual preparatory session based on treatment assignment (described below).

### 5.4 Inclusion/Exclusion Criteria

**Inclusion Criteria:**
Eligible Veterans are age 60 to 95 years old and have had at least 3 months of musculoskeletal pain, including the following conditions most likely to benefit from psychosocial intervention based on previous research (28):

- regional pain syndromes (e.g., low back, neck, leg, or pelvic pain, or temporomandibular joint disorders);
- widespread pain syndromes (e.g., fibromyalgia);
- whiplash;
- tension headaches; or
- any combination of these disorders.

**Exclusion Criteria:**
We exclude the following conditions:

- Confirmed hip or knee osteoarthritis without other musculoskeletal pain complaints;

- Leg pain greater than back pain only without other musculoskeletal pain complaints (to exclude radiculopathy in isolation);
- EMG-confirmed “tunnel” syndromes (e.g., carpal or tarsal tunnel syndrome) only without other musculoskeletal pain complaints;
- Autoimmune disease that typically generates pain (e.g., systemic lupus erythematosus, rheumatoid arthritis, inflammatory bowel disease, ankylosing spondylitis);
- Cancer pain, sickle cell disease, neuralgias (e.g., trigeminal neuralgia), burn pain, infection associated with pain (e.g., vertebral infection), cauda equina syndrome, gout, migraine or cluster headaches without additional musculoskeletal pain complaints;
- Uncontrolled severe psychiatric disorder including current psychotic disorder or severe mood disorder not controlled with medications (e.g., schizophrenia or bipolar I disorder), dissociative identity disorder, or active suicide/violence risk in the past 6 months;
- Substantial cognitive impairment or dementia (Mini-Mental State Exam (78) score ≤ 24);
- Active severe alcohol or substance use disorder that inhibits the participant’s ability to attend session or participate in homework;
- Currently with pain-related litigation or applying for pain-related compensation or compensation increase, or undergoing compensation re-evaluation (e.g., applying for VA service connection or service connection increase for pain);
- Patients who are currently receiving CBT or EAET psychotherapy treatment for pain;
- Patients who have received CBT for pain in the past 3 months;
- Unable to fluently read/converse in English;
- Planning to move from the area in the next 6 months.

**Medications/Other Treatments:**
Consistent with other trials of psychosocial interventions for chronic pain (23, 24), participants will not be excluded due to using any medications at any dose, or for pursuing other non-psychotherapy treatment options for chronic pain (e.g., trigger point injections, physical therapy), or for participating in psychotherapy for another condition (e.g., depression, insomnia). However, current medications and dosages, other treatments, and presence of psychotherapy for other conditions are recorded at baseline, post-treatment, and follow-up visits for monitoring purposes and possible post-hoc analyses.

### 5.5 Study Evaluations

Data are collected from participant self-report before, after, and at 6-month follow-up from interventions. Descriptive measures collected at baseline include demographics (age, sex, race/ethnicity, marital status, education) and clinical characteristics (medical comorbidities, medications, psychiatric diagnoses, substance use). Mini-Mental State Examination is collected at screening.

**Primary Outcome:** The primary outcome is change in mean pain severity scores on the Brief Pain Inventory (BPI) (70) from baseline to post-treatment. The BPI is a short, self-administered questionnaire that was developed for use in cancer patients but is now one of the most widely used tools for assessing a variety of pain conditions in clinical and research settings (71, 72). The BPI contains four items rated 0-10 assessing worst and least pain during the last seven days, as well as average pain and current pain. The mean of these four items is determined to assess mean pain severity. This metric is referred to alternately in the literature as “mean pain severity” and the “pain severity index” (24).

**Secondary Outcomes:** Consistent with recommendations from IMMPACT (73) and our pilot data, we examine pain interference, physical symptoms (sleep disturbance, fatigue), emotional symptoms (anxiety, depression, PTSD symptoms), purpose and meaning, patient-reported global impression of change, and satisfaction with treatment as secondary outcomes. PROMIS and NIH Toolbox measures are used, where available, because of their long-term potential to improve standardization of patient-reported outcomes (79).

**Potential Moderators:** Based on previous literature and our pilot work, we examine whether greater baseline anxiety or depression predict greater response to EAET on primary and secondary outcomes post-treatment and at follow-up, and lower baseline anxiety or depression predict greater response to CBT on primary and secondary outcomes post-treatment and at follow-up. We use the same PROMIS measure to evaluate baseline anxiety and depression as used for secondary outcomes.

**Potential Mediators:** Potential mediators are assessed using standardized assessments which have been validated in the chronic pain population. Emotional approach coping, ambivalence over emotional expression, emotion differentiation, self-conscious affect, anger discomfort, and emotional associations are assessed at baseline, post-treatment, and 6-month follow-up to represent emotion regulation, the proposed distinct mediator for EAET. Pain catastrophizing and coping skills are assessed at baseline, post-treatment, and 6-month follow-up to representing cognitive and behavioral coping, the proposed distinct mediators for CBT. Change on these measures baseline to post-treatment and 6-month-follow-up will be compared to changes in outcomes from baseline to post-treatment and 6-month follow-up. In addition, working alliance, pain beliefs, pain attitudes, and pain control are assessed to mediate response to both treatments. Working alliance will be compared to treatment response at post-treatment and 6-month follow-up. Patients will also be given the opportunity to answer a few open-ended questions at 6-month follow up to assess for mediators that may be worth exploring in future research.

All study variables and their associated measurement instruments are shown in **Table 2**.

| **Table 2: Study Assessments** | | | | Base-line | Post-treat. | 6-mo. f/u | Expect. Direct. of Change |
|---|---|---|---|---|---|---|---|
| | **Variable** | **Measurement Instrument** | | | | | |
| **Primary Outcome** | Cognition | Mini-Mental State Examination | | Screening only | | | |
| | Pain Severity | Mean of 4 Brief Pain Inventory (BPI) (70) Items: | Current Pain | √ | √ | √ | ↓ |
| | | | Average Pain | | | | |
| | | | Worst Pain – 7 days | | | | |
| | | | Least Pain – 7 days | | | | |
| **Secondary Outcomes** | Pain Interference | PROMIS-Pain Interference Short Form (80, 81) | | √ | √ | √ | ↓ |
| | Pain Behavior | PROMIS-Pain Behavior Short Form (80, | | √ | √ | √ | ↓ |
| | Depression | PROMIS-Depression Short Form (40) | | √ | √ | √ | ↓ |
| | Anxiety | PROMIS-Anxiety Short Form (40) | | √ | √ | √ | ↓ |
| | PTSD Symptoms | PTSD Symptom Scale (82) | | √ | √ | √ | ↓ |
| | PTSD Symptoms | PCL-5 with LEC-5 and Criterion A (94) | | √ | √ | √ | ↓ |
| | Purpose and | NIH Toolbox-Purpose and Meaning Short | | √ | √ | √ | ↑ |

| | | | | | | |
|---|---|---|---|---|---|---|
| | Meaning | Form (83) | | | | |
| | Life Satisfaction | NIH Toolbox-Life Satisfaction Short Form | √ | √ | √ | ↑ |
| | Positive Affect | NIH Toolbox-Positive Affect Short Form | √ | √ | √ | ↑ |
| | Sleep Disturbance | PROMIS-Sleep Disturbance Short Form (84) | √ | √ | √ | ↓ |
| | Fatigue | PROMIS-Fatigue Short Form (85) | √ | √ | √ | ↓ |
| | Global Improvement | Patient Global Impression of Change (PGIC) (86) | | √ | √ | ↑ |
| | Patient Satisfaction | Satisfaction with Therapy and Therapist Scale—Revised (74) | | √ | | |
| **Mediators** | Ambivalence | Ambivalence over Emotional Expression Questionnaire (AEQ) (46) | √ | √ | √ | ↓ |
| | Emotional Approach | Emotional Approach Coping Scales (EAC) (77) | √ | √ | √ | ↑ |
| | Emotion Differentiation | PANAS Emotion Differentiation (95) | √ | √ | √ | ↑ |
| | Self-Conscious Affect | Test of Self-Conscious Affect 3 - short version (TOSCA-3) (96) | √ | √ | √ | ↓ |
| | Anger Discomfort | Anger Discomfort Scale (97) | √ | √ | √ | ↓ |
| | Emotional Associations | Property Association Task (98) | √ | √ | √ | ↑ |
| | Catastrophizing | Pain Catastrophizing Scale (PCS) (87) | √ | √ | √ | ↓ |
| | Pain Coping | Coping Strategies Questionnaire (CSQ) (88) | √ | √ | √ | ↑ |
| | Pain Beliefs | Pain Beliefs and Perceptions Inventory (PBAPI) | √ | √ | √ | ↓ |
| | Pain Control | Beliefs in Pain Control Questionnaire (BPCQ) | √ | √ | √ | ↑ |
| | Pain Attitudes | Survey of Pain Attitudes (SOPA) | √ | √ | √ | ↑ |
| | Working Alliance | Working Alliance Inventory (WAI) (89) | | √ | | |
| | Future Mediators | Open-Ended Questions | | | √ | N/A |

### 5.6 Interventions and Interventionists

**Interventions:**

**Emotional Awareness and Exposure Therapy (EAET)** includes one individual preparatory session plus 8 weekly group therapy sessions for small groups of 8 Veterans. The conceptual model of EAET is based on 1) neurobiological research indicating the importance of the central nervous system in chronic pain disorders; 2) neurobiological evidence showing structural and functional relationships between emotional brain circuits (e.g., anxiety) and brain circuits dealing with pain; 3) experiential and psychodynamic theories and particularly the relationship between life stress, emotional coping, and symptoms (pain); 4) epidemiological evidence relating trauma/life stress to pain; and 5) research relating alexithymia, emotional expression, and emotional awareness to pain and other somatic disorders. We will use the most updated version of the EAET treatment manual and accompanying patient handouts (26). The sessions include:

- Individual preparatory session. Following screening and consent, each participant receives a psychodiagnostic interview (90) and pain history (26). These components focus on stressful events, how the patient unconsciously avoids emotional experiences,

and how this avoidance is linked to the onset and maintenance of pain and associated symptoms (e.g., depression, anxiety, fatigue).

- Group Session 1: Rationale; Model of Stress, Emotions, and Pain; Identifying Stress-Symptom Connections. The therapist presents the intervention and rationale, including the links between life stress, emotional avoidance, and pain, and the role of altered pain pathways in the central nervous system. The therapist presents the task of therapy, which involves attenuating pain through directly addressing stress and experiencing previously avoided emotions. Home exercises throughout will include increasing activities, reading, and completing worksheets on avoidance, stress, and disclosure of emotions.
- Group Session 2: Triangle Model of Stress and Symptoms; Experiencing and Expressing Anger and Closeness. This session focuses on the Triangle Model, which represents how people function internally, including their emotions, avoidance techniques, and anxiety/symptoms. In-session experiential exercises are introduced to focus on overcoming avoidance techniques and fully experiencing and expressing avoided emotions, starting with anger and emotions related to closeness with others. An avoidance log is introduced for patients to keep track of avoided emotional experiences.
- Group Session 3: Avoidance in Daily Life; Conflicted Relationships; and Experiencing, Expressing, and Releasing 1. Conflicted relationships that lead to emotional avoidance are discussed, and imaginal exercises which focus on experiencing, expressing, and releasing difficult feelings during the session are practiced.
- Group Session 4: Reversing Self-Blame and Guilt with Self-Compassion and Self-Forgiveness; Experiencing, Expressing, and Releasing 2. This session focuses on the difference between healthy guilt when one has done wrong and self-blame which is unhealthy and contributes to symptoms. In session exercises focus on reversing self-blame with assertion and building forgiveness and compassion toward oneself.
- Group Session 5: Forgiving Others or Letting Go; Experiencing, Expressing, and Releasing 3. This session focuses on experiences of being violated, hurt, or neglected and releasing emotions that come from these experiences. Further in-session exercises on experiencing, expressing, and releasing emotions are practiced.
- Group Session 6: Shame, Intimacy, and Private Experiences or Secrets. Shame is defined as a sense of self that is bad and discussed as always unhealthy. Experiential exercises focus on intimacy and sharing secrets of which patients are ashamed.
- Group Session 7: Healthy Communication in Relationships. The two parts of healthy communication, connection to others and assertiveness, are discussed and practiced in session. This is distinguished from the intense emotional expression we have practiced thus far.
- Group Session 8: Review and Planning. The final session reviews all exercises and progress made. Participants describe optimal emotional and interpersonal functioning, which serves as a goal for their continued work, and they develop a written plan for continued exercises to perform to meet that goal.

**Cognitive Behavior Therapy (CBT)** also includes one individual preparatory session and 8 group therapy sessions for groups of 8 Veterans. The conceptual model for CBT for chronic pain is that pain is enduring and requires ongoing coping to control or manage it. Patients are taught skills to modify their behaviors and cognitions, which is thought to reduce pain, dysfunction, and distress. Our treatment manual and patient handouts for group CBT are from the VA CBT for Chronic Pain (CBT-CP) Evidence-Based Psychotherapy manual. Sheryl Osato, PhD, (SO) our chief CBT interventionist, adjusted the timing of CBT-CP sessions in order to

match the EAET intervention with respect to the number of sessions, length of sessions, timing of sessions, and amount of homework. SO’s adjustments are described here:

- Individual preparatory session (CBT-CP Manual Session 1). This session reviews the participant’s pain and health history; including medical, behavioral, and other approaches the participant has tried to manage pain. Therapist and patient discuss the outcomes of those efforts and prepare for cognitive and behavioral skills training.
- Group Session 1: Treatment Orientation (CBT-CP Manual Sessions 2-3). The therapist presents the rationale for CBT pain coping skills, which shows that pain is a complex experience influenced by thoughts, feelings, and behaviors.
- Group Session 2: Relaxation Training (CBT-CP Manual Session 5). This session presents relaxation training and demonstrates that relaxation can reduce pain by having patients engage in a moderate pain-eliciting activity both before and after relaxation. Difficulties with relaxation are examined.
- Group Session 3:  Applied Relaxation (CBT-CP Manual Session 4). This session focuses on teaching and implementing applied relaxation (brief relaxation used in the natural environment). The ability to integrate relaxation exercises into one’s daily routine is of paramount importance and will be addressed here.
- Group Session 4: Pleasant Activity Scheduling (CBT-CP Manual Sessions 6-7). This session encourages pleasant activities, which can combat negative mood. Activity pacing (identifying activities in which patients overexert themselves and then dividing activities into periods of activity and rest) is taught to avoid pain flares.
- Group Sessions 5-6: Cognitive Coping and Distraction (CBT-CP Manual Sessions 8-9). These sessions focus on changing cognitions underlying pain, particularly catastrophizing. Cognitive techniques are taught to help patients recognize relationships among thoughts, feelings, and behaviors and replace maladaptive thoughts with alternative, rational coping thoughts. Relaxation through distraction techniques are also taught.
- Group Session 7: Sleep (CBT-CP Manual Session 10). This session addresses the importance of sleep as it relates to chronic pain. Sleep hygiene will be reviewed with patients, and exercises will be completed to improve sleep hygiene.
- Group Session 8 (CBT-CP Manual Session 11). Review and Planning. This session focuses on planning for maintenance by having each participant develop a written plan that lists coping skills to deal with setbacks.

**Interventionists:**

Experienced interventionists will be primarily responsible for the administration of both EAET and CBT, in keeping with the primary efficacy aim of this research. Hannah Roggenkamp, MD, will conduct EAET sessions, and Sheryl Osato, PhD, will conduct CBT interventions.

As they become available, trainees are recruited to assist the primary interventionists with administration of EAET and CBT. Trainee interventionists are psychologists with at least a master’s degree in clinical psychology (licensed psychologists, psychology interns, or psychology residents), psychiatrists or psychiatry residents who have completed at least two years of post-graduate training, licensed marriage and family therapists, or social workers. Each interventionist conducts only one of the two conditions to preserve fidelity by assuring there is no overlap or blending of treatments or allegiance effects.

All interventionists receive a detailed outline of the protocol and participate in exercises, activities and group management skills taught through didactic instruction, taped illustrations from model cases, and role-plays of common scenarios prior to assisting with interventions.

Trainee interventionists shadow and assist the primary interventionists for up to eight sessions before conducting interventions independently. Supervision occurs weekly for trainee interventionists working independently, in which video recordings of sessions are reviewed, and it is verified that the skills and content are being faithfully presented according to protocol. Primary interventionists make ratings of adherence to the protocols to assess trainees. Numerical protocol adherence criteria are developed for each session, with satisfactory adherence defined as ≥90% of the maximum possible score on the adherence rating scale. Training is reinitiated when adherence is found to decrease below 90% (91). In addition, independent fidelity monitoring occurs for a randomly selected 25% of all sessions (see section below).

**Fidelity Monitoring:**
Both EAET and CBT therapy sessions are video recorded for the purposes of fidelity monitoring. Video recording is essential for EAET, given the importance of observing participants' nonverbal behavior for this intervention. An independent fidelity monitor, who is a geriatric psychiatrist with research experience, reviews the video recording of one early and one late session from each course of group EAET or CBT (2 out of every 8 sessions = 25%). In addition, the fidelity monitor reviews 2 out of every 8 = 25% individual psychotherapy sessions for both EAET and CBT. The fidelity monitor completes a standardized checklist to ensure that the session covered targeted topics (based on therapy model) but did not contain non-targeted topics (e.g., CBT concepts during EAET treatment). Participants consent to the production of video recording at the time of informed consent for the study by completing HIPAA authorization, in compliance with IRB policy. Videos are produced on a VA-approved laptop, and video files are immediately transferred to our encrypted VA research server.

### 5.7 Data Analysis

**Statistical Plan:**

The experimental design for this study is a 2-group randomized superiority trial, with assessments at baseline, post-treatment (primary endpoint), and 6-month follow-up. The aims correspond to comparisons of the effects of two active treatments to each other (Aim 1), moderation of the interventions (Aim 2), and mediation of the interventions (Aim 3).

Prior to performing the primary analyses, descriptive statistics and graphical summaries are obtained for all outcomes to check for missing data, outliers, and the need for transformations or non-parametric methods.

To assess the success of randomization, continuous baseline covariate statistics will be reported and compared with p-values computed using the t-test or the nonparametric analog (Wilcoxon rank sum test). The p-values for comparing categorical baseline covariates will be computed using chi-square tests or the nonparametric analog (Fisher's exact test). We will compare primary pain and secondary outcomes, demographic (age, race/ethnicity, marital status, education) and clinical (number of medical conditions, presence of psychiatric disorder, presence of substance use, presence of opioid medications) characteristics. While will do not anticipate imbalance in a randomized trial, baseline factors that are clinically and statistically significant and are thought or shown to have a nontrivial effect on the pain change or other outcomes will be considered as adjustment factors. If adjustments are needed, both unadjusted and adjusted results will be reported.

Aim 1: Treatment Group Comparisons: The general linear mixed model (GLMM) will be used to compare BPI mean pain severity changes from baseline and other secondary continuous outcomes between the two groups (EAET, CBT) over time in order to account for correlations induced by repeated measures within subjects and allow for both fixed and time-varying covariates. For ease of interpretation, change from baseline is used as the outcome instead of modeling post-treatment pain with baseline pain as an additional predictor. The GLMM produces consistent estimates when there are missing data if the data are missing at random (MAR). Group, time, and the group x time interaction will be evaluated accounting for correlations via random person effects. Analyses will be carried out via intent to treat (ITT). The primary analyses will use all data.

If an adjusted analysis is needed, we will use logistic regression of treatment group versus the selected covariates to create a propensity score. This score will then be used as an added covariate in the GLMM or will be used to create adjustment strata if the association of pain change with the propensity score is nonlinear or not easily modelled.

Aim 2: Moderation of the Interventions. We hypothesize that patients with higher baseline depression and anxiety will experience greater pain reduction from EAET relative to CBT, whereas those with lower baseline depression and anxiety will benefit more from CBT relative to EAET. To test moderation of condition effects by these baseline characteristics, we will use the PROCESS macro developed by Hayes (92). This approach, which is based on the most current statistical considerations for model testing, involves bootstrapped analyses and estimates of various prespecified models, including tests of both moderators and mediators (92). We will examine depression and anxiety separately to test our hypotheses, but we will also explore, using principal components analysis, whether depression and anxiety should be combined into a composite. Significant interactions will be plotted for post-hoc probing, and high and low values (e.g., ± 1 SD from the sample mean) will be plotted. We also will examine cut-scores on the moderators to classify participants into subgroups that are responsive or amenable to each intervention—an approach that can facilitate later tailoring or treatment matching. For exploratory purposes, we will carry out the same analyses for the secondary outcome measures as well.

Aim 3 (Exploratory): Mediation of the Interventions. This aim pertains to the potential mediating effects of ambivalence over emotional expression and emotional approach coping on the effects of EAET, of pain coping and catastrophizing on CBT, and working alliance on both EAET and CBT. For primary and secondary outcomes at post-treatment, we will test the potential mediators that are assessed concurrently. For the 6-month follow-up evaluation, we will test both the post-treatment and the 6-month follow-up (concurrent) mediators. We will examine the mediators separately, but we also will use multivariate methods (principle component scoring algorithm) to explore whether the potential mediators for each intervention should be combined into composites. We will assess mediation by also using the PROCESS macro developed by Hayes (92, 93).

**Sample Size and Power Considerations:**

Aim 1: Data from our first two pilot groups showed a mean 1.13-point improvement in BPI mean pain severity under EAET compared to a 0.33-point mean improvement of BPI mean pain severity under CBT, a mean difference of 1.13 – 0.33 = 0.80 points. Using a sample size of n = 60 per group and assuming the same SDs, there is 80% power to confirm mean differences as small as 0.70 points using the usual two-sided $\alpha = 0.05$ significance level. In our pilot, only six of 32 participants were lost to follow-up after

completing baseline assessments and randomization. We estimate at least this dropout rate for the proposed study and possibly up to 25% of participants (based on previous studies, e.g., (16)). We therefore will need to enroll at least 60/0.75 = 80 participants per group or 160 participants total.

Aim 2: A sample size of n = 60 has 80% power ($\alpha$=0.05) for confirming correlations as small as r = 0.35 (in absolute value) using the usual $\alpha$ = 0.05 significance level. Our pilot data shows that the correlation between anxiety and pain change in the EAET group was r = -0.40, and the correlation between anxiety and pain change in the CBT group was r = 0.53, both well within this margin. Based on the findings of Burger, et al. (23), we anticipate a possibly larger correlation between depression and reduction in pain than found in our pilot groups. We believe that correlations between pain severity change and depression and anxiety also may be even larger, given the large variation in the pilot sample.

**Dropouts:**

While we anticipate few dropouts, we will use a logistic or Cox proportional hazard regression model to assess the association between baseline covariates including the demographic and clinical covariates, versus dropping out or time to drop out. If the dropouts are missing completely at random (MCAR), there should be no association between any of the baseline covariates and dropping out. If the dropouts are MCAR, they are MAR. If the dropouts are not MCAR, we will carry out secondary subanalyses for baseline and post-treatment using only the subset that completed both and secondary subanalyses for baseline, post treatment and six-month follow up using only those who completed all three. Based on comparing this to the primary analysis using all data, we will consider sensitivity analyses to try to quantify the amount of possible bias.

**Personnel and Location for Analyses:**

Statistical analyses will be conducted primarily by the PI in his office in Bldg. 401, Rm. A236 or in the Brain, Behavior, and Aging Research Center in Bldg. 158 Rms. 154-169 on VA computers with statistical software including STATA and SPSS.

### 5.8 Withdrawal of Subjects

Although extremely unlikely, it is possible that a participant's participation in the study might be terminated by the investigator without regard to their consent. Instances in which termination could occur include excessive absences (>4) from psychotherapy sessions or extreme disruptiveness in sessions. The participant would be notified of termination in advance and in writing.

No adverse effects of termination from this study would be anticipated. However, the participant would not be likely to receive the benefits of full participation described above.

If you a participant is withdrawn from the study, no additional follow-up would be required. However, any data already collected prior to withdrawal from the study may be used by the investigators. No further information will be collected after a participant's withdrawal from the study.

## 6.0 Reporting/Risks/Safety

**Reporting:**

In the event of an unanticipated problem, serious adverse event, or protocol deviation, this information will be reported immediately to the IRB according to IRB policy by submitting a Form 5.

In addition, data and safety monitoring for this study will be provided by the Clinical Science Research & Development (CSR&D) centralized Data Monitoring Committee (DMC). The DMC is provided by CSR&D to ensure independent oversight of the safety and integrity of the project. The DMC is an independent multidisciplinary group, whose members have collectively – through research, education, training, experience, and expertise – the requisite knowledge pertinent to the subject areas to be reviewed. Membership details are available on the CSRD website. The DMC will provide an ongoing independent evaluation of this study focused on safety and feasibility, including participant accrual and retention, adverse events monitoring, and data analyses. Meetings will be held three times per year at which recommendations will be made to the Director of CSR&D for endorsement. These recommendations will range from approval to continue (unconditionally or with conditions to be addressed) to probation or possibly termination, if there are problems with enrollment or safety concerns.

**Risks and Risk Minimization:**

Clinical interview, psychotherapy interventions, and questionnaires: no major risks. Minor risks include inconvenience, frustration with questions, sadness or distress with discussing emotional experiences, and potential embarrassment if the confidentiality of research records is breached. We minimize breach of confidentiality by keeping all participant data in a locked cabinet. Regarding psychotherapy specifically, participants may feel discomfort disclosing personal information to the therapist and other group members. Participants are also at risk for nonresponse or limited response to treatment but are always free to end participation in the study and pursue alternate treatment if they so choose.

We will be monitoring for safety of participants throughout the study. At the beginning of each therapy session, there will be a verbal check-in period in which patients can discuss any worsening of symptoms, or any suicidal ideation. In addition, in case of worsening of symptoms between sessions, the participant will be provided with the PI's direct telephone number (424-279-8439), in addition to the telephone number for his/her therapist and other study personnel, and the participant will be encouraged to call.

On check-in or upon receiving a phone call from the participant, if the participant is found to have worsening of physical medical symptoms (e.g., pain), the patient's primary care provider will be contacted, and a close follow-up appointment will be obtained for the participant. In the event of worsening psychiatric symptoms (e.g., depression, anxiety), the patient may be referred to their mental health provider, or a referral may be placed to the Geriatric Psychiatry Outpatient Program in the even the participant does not have a primary mental health provider. In the event of suicidal or violent ideation, the participant will be referred immediately to the emergency department or to 911, as outlined in our suicide safety plan below.

In the event of worsening physical or medical symptoms, study procedures (i.e., psychotherapy) will be suspended after a conversation with the participant, if that is the

participant’s preference. In the event of suicidal or violent ideation, study procedures (i.e., psychotherapy) will be suspended immediately.

**Suicide Safety Plan:**

We do not expect any study participants to develop suicidal ideation in this treatment study for chronic pain. However, it is known that chronic pain patients have twice the rate of suicide of those without chronic pain. Therefore, we have a suicide safety plan in place. Research physicians and the research psychologist are clinicians in geriatric psychiatry, and each session will begin with a verbal check-in period, and assessments will be completed periodically to monitor for depression. If researchers feel that the participant is at risk for suicide and/or a threat to others while he/she is present at WLA either undergoing clinical interview, completion of assessments, therapy sessions, or other study procedures, he/she will be evaluated by a trained research physician or psychologist who is a study team member and brought to the WLA ER if psychiatric hospitalization is thought to be required. All Veterans will have the PI’s direct telephone number (424-279-8439) to call in the event of worsening symptoms and will also be instructed to dial 911 or report to the nearest ER if a psychiatric emergency, such as suicidal or violent ideation, occurs during the study but while the Veteran is not present at WLA. If a Veteran participant is noted to have significant depression but is not suicidal, the research team will discuss the benefits of treatment and provide contact information for the GLA Geriatric Psychiatry Outpatient Program.

**Potential Benefits:**

The study will be of direct relevance and benefit to older Veterans who suffer from chronic pain. All study participants will receive a psychological intervention, either standard treatment (CBT) or a promising experimental treatment (EAET). We anticipate this could be of benefit for participants on pain and related outcomes, such as mood, anxiety, sleep, and fatigue. Results from the study will inform future research on psychological interventions for chronic pain and contribute to understanding the psychological change processes involved in improvements in chronic pain.

**Risk/Benefit Ratio:**

The risks of this study are small. We will be gaining valuable information to help improve our understanding of the psychological processes involved in improvement in chronic pain and help inform research aimed at using alternate psychological treatment strategies for older adults with chronic pain.

**Alternatives to Participation:**

The patient may receive ongoing medical care in their respective clinics, including in primary care, pain clinic, Women’s Clinic, etc. for pain whether or not s/he participates in the study. This includes medications and interventional pain approaches (e.g. injections, ketamine). The VA also offers a chronic pain rehabilitation program, cognitive behavior therapy for chronic pain, and physical and occupational therapy for chronic pain. Mindfulness classes, acupuncture, and other complementary/integrated medicine approaches are also offered. The patient may elect to not participate, and this will not adversely affect any ongoing care at the VA.

## 7.0 Privacy and Confidentiality

Taking part in this study will involve collecting private information about participants, including name, diagnoses, medications, responses to study questionnaires, and video recordings. All information will be labeled with an alphanumeric code to protect participant confidentiality. Study records will be stored in a locked cabinet in Bldg. 401, Rm. A236 or Bldg. 158 Rm. 154-169. See below for further information on data security.

Information about participants will be combined, publications and presentations about the combined data we have gathered will be created. Any talks or papers about this study will not identify individual participants.

In addition, a description of this clinical trial will be available on http://www.ClinicalTrials.gov as required by U.S. Law. This website will not include PHI for study participants. At most, the website will include a summary of the results.

We will not share participant records or identify participants unless we are legally required. Participants will be notified that the following government agencies may have access to their records: Office of Human Research Protections, the Government Accountability Office, the Office of the Inspector General, the VA Office of Research Oversight, the VA Greater Los Angeles IRB, our local Research and Development Committee, the sponsor (VA Clinical Science Research and Development – CSR&D), and the CSR&D Data Monitoring Committee.

By law, information can still be released if we find or suspect child abuse, elder abuse, an by a participant intent to harm him/herself or others, or if a participant has an infectious disease that State or Federal law requires us to report.

Participants are permitted to release data about themselves or involvement in the study.

## 8.0 Communication Plan

**Data Security Plan:**

At the time of a patient's enrollment, appropriate informed consent is obtained, and all prior identifying information obtained during screening is destroyed. Each participant has a hardcopy of the original signed consent form. A study folder for each participant is created with an alphanumeric code that does not include portions of the participant's social security number. These folders contain completed questionnaires and data sheets with corresponding labeling by alphanumeric code. The decoding algorithm is stored in a password protected encrypted electronic file. Hardcopy study folders are stored in a locked filing cabinet in the locked office of the PI (WLA, Bldg. 401, Rm. A236). The PI has primary access to the key to the filing cabinet where hardcopy records are kept. Other project staff (Study Coordinator, investigators, and assistants) have access to the hardcopy records only through the PI.

For video files of psychotherapy sessions, a customized VA intranet-based data system is created. Data are collected and processed on a separate VA laptop computer, and the files are extracted and uploaded into the primary database. The system is housed on the VAGLAHS Geriatric Psychiatry research server. An alphanumeric code is generated for all video files. All files are encrypted, and the systems and tools are protected by 128-bit SSL, the secure socket layer technology used for sensitive transactions on the web. The system is accessible only via the VA intranet and employs a hierarchical system of password protected logins, allowing differential access to project team members as appropriate to their roles. Data are accessed only through the system, not transported. Dr. Sultzer (Co-Investigator) and his team have extensive experience conducting and

supporting studies ranging from small pilot studies to large multisite centers, which have required creating data systems such as this for neuroimaging data and other types of encrypted electronic files.

**Data Banking/Future Use:**

Participants will be invited to have their data (interviews, questionnaires, and video recordings of psychotherapy sessions) stored in a new repository after study closure for future research involving psychotherapy, aging, psychological processes, and chronic pain; this will be optional for participants. Prior to study closure, an application for the creation of a new data repository will be submitted to the local IRB. Information will be labeled with a code that does not contain participants name or other ways that identify them. Any future research will be done for research purposes only; participants' doctors will not be notified about the results of the research.

**Privacy Breaches:**

In the event of a privacy breach, the IRB and involved participants will be notified immediately, in accordance with IRB policy.

## 9.0 References


1. Kerns RD, Otis J, Rosenberg R, et al. Veterans' reports of pain and associations with ratings of health, health-risk behaviors, affective distress, and use of the healthcare system. Journal of rehabilitation research and development. 2003;40(5):371.
2. Clark JD. Chronic pain prevalence and analgesic prescribing in a general medical population. Journal of pain and symptom management. 2002;23(2):131-7.
3. Haskell SG, Heapy A, Reid MC, et al. The prevalence and age-related characteristics of pain in a sample of women veterans receiving primary care. Journal of Women's Health. 2006;15(7):862-9.
4. Beckham JC, Crawford AL, Feldman ME, et al. Chronic posttraumatic stress disorder and chronic pain in Vietnam combat veterans. Journal of psychosomatic research. 1997;43(4):379-89.
5. Von Korff M, Kolodny A, Deyo RA, et al. Long-term opioid therapy reconsidered. Annals of internal medicine. 2011;155(5):325-8.
6. Stewart MO, Karlin BE, Murphy JL, et al. National dissemination of cognitive-behavioral therapy for chronic pain in veterans: therapist and patient-level outcomes. Clin J Pain. 2015;31(8):722-9. Epub 2014/08/30. doi: 10.1097/ajp.0000000000000151. PubMed PMID: 25171637.
7. Dhalla IA, Persaud N, Juurlink DN. Facing up to the prescription opioid crisis. BMJ: British Medical Journal (Online). 2011;343.
8. Baser O, Xie L, Mardekian J, et al. Prevalence of Diagnosed Opioid Abuse and its Economic Burden in the Veterans Health Administration. Pain Practice. 2014;14(5):437-45. doi: 10.1111/papr.12097.
9. Larney S, Bohnert ASB, Ganoczy D, et al. Mortality among older adults with opioid use disorders in the Veteran's Health Administration, 2000–2011. Drug and Alcohol Dependence. 2015;147:32-7. doi: https://doi.org/10.1016/j.drugalcdep.2014.12.019.
10. Saunders KW, Dunn KM, Merrill JO, et al. Relationship of Opioid Use and Dosage Levels to Fractures in Older Chronic Pain Patients. Journal of General Internal Medicine. 2010;25(4):310-5. doi: 10.1007/s11606-009-1218-z.
11. Group TOTfCPW. VA/DoD Clinical Practice Guideline for Opioid Therapy for Chronic Pain. Washington, DC: VA/DoD, 2017 February 2017.
12. Dowell D, Haegerich TM, Chou R. CDC guideline for prescribing opioids for chronic pain—United States, 2016. Jama. 2016;315(15):1624-45.
13. Karlin BE, Cross G. From the laboratory to the therapy room: National dissemination and implementation of evidence-based psychotherapies in the U.S. Department of Veterans Affairs Health Care System. Am Psychol. 2014;69(1):19-33. Epub 2013/09/05. doi: 10.1037/a0033888. PubMed PMID: 24001035.
14. Williams AC, Eccleston C, Morley S. Psychological therapies for the management of chronic pain (excluding headache) in adults. Cochrane Database Syst Rev. 2012;11:Cd007407. Epub 2012/11/16. doi: 10.1002/14651858.CD007407.pub3. PubMed PMID: 23152245.

15. McCracken LM, Turk DC. Behavioral and cognitive–behavioral treatment for chronic pain: outcome, predictors of outcome, and treatment process. Spine. 2002;27(22):2564-73.
16. Herbert MS, Afari N, Liu L, et al. Telehealth Versus In-Person Acceptance and Commitment Therapy for Chronic Pain: A Randomized Noninferiority Trial. J Pain. 2017;18(2):200-11. Epub 2016/11/14. doi: 10.1016/j.jpain.2016.10.014. PubMed PMID: 27838498.
17. Wetherell JL, Afari N, Rutledge T, et al. A randomized, controlled trial of acceptance and commitment therapy and cognitive-behavioral therapy for chronic pain. Pain. 2011;152(9):2098-107. Epub 2011/06/21. doi: 10.1016/j.pain.2011.05.016. PubMed PMID: 21683527.
18. McCracken LM, Vowles KE. Acceptance and commitment therapy and mindfulness for chronic pain: Model, process, and progress. American Psychologist. 2014;69(2):178.
19. Walser RD, Garvert DW, Karlin BE, et al. Effectiveness of Acceptance and Commitment Therapy in treating depression and suicidal ideation in Veterans. Behav Res Ther. 2015;74:25-31. Epub 2015/09/18. doi: 10.1016/j.brat.2015.08.012. PubMed PMID: 26378720.
20. Hann KE, McCracken LM. A systematic review of randomized controlled trials of Acceptance and Commitment Therapy for adults with chronic pain: Outcome domains, design quality, and efficacy. Journal of Contextual Behavioral Science. 2014;3(4):217-27.
21. Chiesa A, Serretti A. Mindfulness-based interventions for chronic pain: a systematic review of the evidence. J Altern Complement Med. 2011;17(1):83-93. Epub 2011/01/27. doi: 10.1089/acm.2009.0546. PubMed PMID: 21265650.
22. Veehof MM, Oskam M-J, Schreurs KM, et al. Acceptance-based interventions for the treatment of chronic pain: a systematic review and meta-analysis. PAIN®. 2011;152(3):533-42.
23. Burger AJ, Lumley MA, Carty JN, et al. The effects of a novel psychological attribution and emotional awareness and expression therapy for chronic musculoskeletal pain: A preliminary, uncontrolled trial. Journal of psychosomatic research. 2016;81:1-8.
24. Lumley MA, Schubiner H, Lockhart NA, et al. Emotional awareness and expression therapy, cognitive behavioral therapy, and education for fibromyalgia: a cluster-randomized controlled trial. Pain. 2017;158(12):2354-63.
25. Abbass A, Kisely S, Kroenke K. Short-term psychodynamic psychotherapy for somatic disorders. Psychotherapy and psychosomatics. 2009;78(5):265-74.
26. Lumley MA, Schubiner H. Emotional Exposure Therapy for Stress Reduction: A Group-Based Treatment Manual for Patients with Fibromyalgia and Related Chronic Pain Disorders (Version 2). 2012.
27. Lumley M, Schubiner H, Clauw D, et al. The PAST-FM (Pain and Stress Treatment for Fibromyalgia) randomized, controlled trial: main effects of emotional awareness and expression and cognitive-behavioral therapies. J Pain. 2016;17(4S):S102. doi: 10.1016/j.jpain.2016.01.316. PubMed PMID: 28162316.
28. Clauw DJ. Diagnosing and treating chronic musculoskeletal pain based on the underlying mechanism (s). Best Practice & Research Clinical Rheumatology. 2015;29(1):6-19.
29. Kosek E, Cohen M, Baron R, et al. Do we need a third mechanistic descriptor for chronic pain states? Pain. 2016;157(7):1382-6.
30. Hashmi JA, Baliki MN, Huang L, et al. Shape shifting pain: chronification of back pain shifts brain representation from nociceptive to emotional circuits. Brain. 2013;136(9):2751-68.
31. Erikson EH. Identity and the life cycle: Selected papers. Psychological issues. 1959.

32. Gallagher-Thompson D, Steffen AM. Comparative effects of cognitive-behavioral and brief psychodynamic psychotherapies for depressed family caregivers. J Consult Clin Psychol. 1994;62(3):543-9. Epub 1994/06/01. PubMed PMID: 8063980.
33. Thompson LW, Gallagher D, Breckenridge JS. Comparative effectiveness of psychotherapies for depressed elders. Journal of consulting and clinical psychology. 1987;55(3):385.
34. Raue PJ, Schulberg HC, Heo M, et al. Patients' depression treatment preferences and initiation, adherence, and outcome: a randomized primary care study. Psychiatr Serv. 2009;60(3):337-43. doi: 10.1176/appi.ps.60.3.337. PubMed PMID: 19252046; PubMed Central PMCID: PMCPMC2710876.
35. Gum AM, Areán PA, Hunkeler E, et al. Depression Treatment Preferences in Older Primary Care Patients. The Gerontologist. 2006;46(1):14-22.
36. McWilliams LA, Cox BJ, Enns MW. Mood and anxiety disorders associated with chronic pain: an examination in a nationally representative sample. 2003;106(1-2):127-33.
37. Tunks ER, Crook J, Weir RJ. Epidemiology of chronic pain with psychological comorbidity: prevalence, risk, course, and prognosis. 2008;53(4):224-34.
38. Tsang A, Von Korff M, Lee S, et al. Common chronic pain conditions in developed and developing countries: gender, age differences and comorbidity with depression-anxiety disorders. 2008;9(10):883-91.
39. Ozomaro U, Wahlestedt C, Nemeroff CB. Personalized medicine in psychiatry: problems and promises. BMC medicine. 2013;11(1):132.
40. Pilkonis PA, Choi SW, Reise SP, et al. Item banks for measuring emotional distress from the Patient-Reported Outcomes Measurement Information System (PROMIS(R)): depression, anxiety, and anger. Assessment. 2011;18(3):263-83. doi: 10.1177/1073191111411667. PubMed PMID: 21697139; PubMed Central PMCID: PMCPMC3153635.
41. Lane RD, Ryan L, Nadel L, Greenberg L. Memory reconsolidation, emotional arousal, and the process of change in psychotherapy: New insights from brain science. Behav Brain Sci. 2015;38:e1. doi: 10.1017/S0140525X14000041. PubMed PMID: 24827452.
42. Taylor GJ, Bagby RM, Parker JD. Disorders of affect regulation: Alexithymia in medical and psychiatric illness: Cambridge University Press; 1999.
43. Davis MC, Zautra AJ, Smith BW. Chronic pain, stress, and the dynamics of affective differentiation. 2004;72(6):1133-60.
44. Borkovec TD, Roemer L, Kinyon J. Disclosure and worry: Opposite sides of the emotional processing coin. 1995.
45. Kennedy-Moore E, Watson JC Expressing emotion. NY: 1999.
46. King LA, Emmons RA. Conflict over emotional expression: psychological and physical correlates. J Pers Soc Psychol. 1990;58(5):864-77. Epub 1990/05/01. PubMed PMID: 2348373.
47. Lane JD, Wegner DM. The cognitive consequences of secrecy. APJJoP. 1995;69(2):237.
48. Stanton AL, Danoff-Burg S, Cameron CL, Ellis. Coping through emotional approach: Problems of conceptualizaton and confounding. APJJoP. 1994;66(2):350.
49. Burns JW, Bruehl S, Quartana PJ. Anger management style and hostility among patients with chronic pain: effects on symptom-specific physiological reactivity during anger-and sadness-recall interviews. JPM. 2006;68(5):786-93.

50. Burns JW, Quartana P, Gilliam W, et al. Effects of anger suppression on pain severity and pain behaviors among chronic pain patients: Evaluation of an ironic process model. APJJoP. 2008;27(5):645.
51. Quartana PJ, Burns JW. Painful consequences of anger suppression. Emotion. 2007;7(2):400.
52. Kross E, Berman MG, Mischel W, et al. Social rejection shares somatosensory representations with physical pain. Proceedings of the National Academy of Sciences. 2011;108(15):6270-5.
53. Sarno JE. Chronic back pain and psychic conflict. JSJoRM. 1976;8(3-4):143-53.
54. Sarno JE. Psychosomatic backache. JTJofp. 1977.
55. Frattaroli J. Experimental disclosure and its moderators: a meta-analysis. JPb. 2006;132(6):823.
56. Smyth JM. Written emotional expression: effect sizes, outcome types, and moderating variables. JJoc, Psychology C. 1998;66(1):174.
57. Norman SA, Lumley MA, Dooley JA, et al. For whom does it work? Moderators of the effects of written emotional disclosure in a randomized trial among women with chronic pelvic pain. Psychosomatic Medicine. 2004;66(2):174-83.
58. Graham JE, Lobel M, Glass P, et al. Effects of written anger expression in chronic pain patients: making meaning from pain. JJobm. 2008;31(3):201-12.
59. Broderick JE, Junghaenel DU, Schwartz JE. Written emotional expression produces health benefits in fibromyalgia patients. JPM. 2005;67(2):326-34.
60. Keefe FJ, Van Horn Y. Cognitive-behavioral treatment of rheumatoid arthritis pain maintaining treatment gains. Arthritis & Rheumatology. 1993;6(4):213-22.
61. Okifuji A, Ackerlind S. Behavioral medicine approaches to pain. Anesthesiology clinics. 2007;25(4):709-19.
62. Jensen KB, Kosek E, Wicksell R, et al. Cognitive Behavioral Therapy increases pain-evoked activation of the prefrontal cortex in patients with fibromyalgeia. 2012;153(7):1495-503.
63. Seminowicz DA, Shpaner M, Keaser ML, et al. Cognitive-behavioral therapy increases prefrontal cortex gray matter in patients with chronic pain. 2013;14(12):1573-84.
64. Krupnick JL, Sotsky SM, Simmens S, et al. The role of the therapeutic alliance in psychotherapy and pharmacotherapy outcome: findings in the National Institute of Mental Health Treatment of Depression Collaborative Research Program. J Consult Clin Psychol. 1996;64(3):532-9. PubMed PMID: 8698947.
65. Karlin BE, Brown GK, Trockel M, et al. National dissemination of cognitive behavioral therapy for depression in the Department of Veterans Affairs health care system: therapist and patient-level outcomes. J Consult Clin Psychol. 2012;80(5):707-18. doi: 10.1037/a0029328. PubMed PMID: 22823859.
66. Thakur E, Holmes H, Lockhart N, et al. Emotional awareness and expression training improves irritable bowel syndrome: A randomized controlled trial. Neurogastroenterology & Motility. 2017;29(12).
67. Keefe FJ, Anderson T, Lumley MA, et al. A randomized, controlled trial of emotional disclosure in rheumatoid arthritis: Can clinician assistance enhance the effects? PAIN®. 2008;137(1):164-72.

68. Lumley MA, Cohen JL, Stout RL, et al. An emotional exposure-based treatment of traumatic stress for people with chronic pain: Preliminary results for fibromyalgia syndrome. Psychotherapy: Theory, Research, Practice, Training. 2008;45(2):165.

69. Hsu MC, Schubiner H, Lumley MA, et al. Sustained pain reduction through affective self-awareness in fibromyalgia: a randomized controlled trial. Journal of general internal medicine. 2010;25(10):1064-70.

70. Tan G, Jensen MP, Thornby JI, et al. Validation of the Brief Pain Inventory for chronic nonmalignant pain. J Pain. 2004;5(2):133-7. Epub 2004/03/26. doi: 10.1016/j.jpain.2003.12.005. PubMed PMID: 15042521.

71. Keller S, Bann CM, Dodd SL, et al. Validity of the Brief Pain Inventory for Use in Documenting the Outcomes of Patients With Noncancer Pain. The Clinical Journal of Pain. 2004;20(5):309-18. PubMed PMID: 00002508-200409000-00005.

72. Cleeland CS, Ryan KM. Pain assessment: Global use of the Brief Pain Inventory. Annals, Academy of Medicine, Singapore. 1994;23(2):129-38.

73. Dworkin RH, Turk DC, Farrar JT, et al. Core outcome measures for chronic pain clinical trials: IMMPACT recommendations. Pain. 2005;113(1-2):9-19.

74. Oei TP, Green AL. The Satisfaction With Therapy and Therapist Scale--Revised (STTS-R) for group psychotherapy: Psychometric properties and confirmatory factor analysis. Professional Psychology: Research and Practice. 2008;39(4):435.

75. Dworkin RH, Turk DC, Wyrwich KW, et al. Interpreting the clinical importance of treatment outcomes in chronic pain clinical trials: IMMPACT recommendations. The Journal of Pain. 2008;9(2):105-21.

76. King LA, Emmons RA. Conflict over emotional expression: psychological and physical correlates. Journal of personality and social psychology. 1990;58(5):864.

77. Austenfeld JL, Stanton AL. Coping through emotional approach: a new look at emotion, coping, and health-related outcomes. Journal of personality. 2004;72(6):1335-64.

78. Folstein MF, Folstein SE, McHugh PR. "Mini-mental state". A practical method for grading the cognitive state of patients for the clinician. J Psychiatr Res. 1975;12(3):189-98. Epub 1975/11/01. PubMed PMID: 1202204.

79. Bevans M, Ross A, Cella D. Patient-Reported Outcomes Measurement Information System (PROMIS): efficient, standardized tools to measure self-reported health and quality of life. Nursing outlook. 2014;62(5):339-45.

80. Askew RL, Kim J, Chung H, et al. Development of a crosswalk for pain interference measured by the BPI and PROMIS pain interference short form. Quality of Life Research. 2013;22(10):2769-76. doi: 10.1007/s11136-013-0398-5.

81. Amtmann D, Cook KF, Jensen MP, et al. Development of a PROMIS item bank to measure pain interference. Pain. 2010;150(1):173-82.

82. Foa EB, Riggs DS, Dancu CV, et al. Reliability and validity of a brief instrument for assessing post-traumatic stress disorder. Journal of traumatic stress. 1993;6(4):459-73.

83. Salsman JM, Lai J-S, Hendrie HC, et al. Assessing psychological well-being: self-report instruments for the NIH Toolbox. Quality of Life Research. 2014;23(1):205-15. doi: 10.1007/s11136-013-0452-3.

84. Yu L, Buysse DJ, Germain A, et al. Development of Short Forms from the PROMIS Sleep Disturbance and Sleep-Related Impairment Item Banks. Behavioral Sleep Medicine. 2011;10(1):6-24. doi: 10.1080/15402002.2012.636266. PubMed PMID: PMC3261577.

85. Cella D, Riley W, Stone A, et al. The Patient-Reported Outcomes Measurement Information System (PROMIS) developed and tested its first wave of adult self-reported health outcome item banks: 2005–2008. Journal of Clinical Epidemiology. 2010;63(11):1179-94. doi: https://doi.org/10.1016/j.jclinepi.2010.04.011.

86. Arnold LM, Lu Y, Crofford LJ, et al. A double-blind, multicenter trial comparing duloxetine with placebo in the treatment of fibromyalgia patients with or without major depressive disorder. 2004;50(9):2974-84.

87. Sullivan MJ, Bishop SR, Pivik J. The pain catastrophizing scale: development and validation. Psychological assessment. 1995;7(4):524.

88. Riddle DL, Jensen MP. Construct and criterion-based validity of brief pain coping scales in persons with chronic knee osteoarthritis pain. JPM. 2013;14(2):265-75.

89. Horvath AO, Greenberg LS. Development and validation of the Working Alliance Inventory. JJocp. 1989;36(2):223.

90. Abbass A. Reaching through resistance: Advanced psychotherapy techniques. Kansas City, MO: 2015.

91. Waltz J, Addis ME, Koerner K, et al. Testing the integrity of a psychotherapy protocol: assessment of adherence and competence. Journal of consulting and clinical psychology. 1993;61(4):620.

92. Hayes A. Introduction to mediation, moderation, and conditional process analysis second edition: A regression-based approach. New York, NY: Guilford Publications, Inc; 2017.

93. Hayes A. Partial, conditional, and moderated moderated mediation: Quantification, inference, and interpretation. FJCM. 2018;85(1):4-40.

94. Blevins CA, Weathers FW, Davis MT, et al. The Posttraumatic Stress Disorder Checklist for DSM-5 (PCL-5): Development and initial psychometric evaluation. Journal of Traumatic Stress. 2015; 28, 489–498. doi: 10.1002/jts.22059

95. Anand D, Chen Y, Lindquist KA, et al. Emotion differentiation predicts likelihood of initial lapse following substance use treatment. Drug Alcohol Dependence. 2017; November 01(180): 439–444. doi: 10.1016/j.drugalcdep.2017.09.007.

96. Turner-Cobb JM, Michalaki M, Osborn M. Self-conscious emotions in patients suffering from chronic musculoskeletal pain: A brief report, Psychology & Health. 2015;30(4): 495-501, doi: 10.1080/08870446.2014.991735

97. Sharkin BS, Gelso CJ. The Anger Discomfort Scale: Beginning reliability and validity data. Measurement & Evaluation in Counseling & Development. 1991; 24(2): 61.

98. MacCormack JK, Henry TR, Davis BM, et al. Aging bodies, aging emotions: Interoceptive differences in emotion representation and report across adulthood. (in press) *Emotion.*
