# Supplementary material for: Emotional Awareness and Expression Therapy vs Cognitive Behavioral Therapy for Chronic Pain in Older Veterans: A Randomized Clinical Trial
Source: JAMA Netw Open. 2024 Jun 13;7(6):e2415842. doi: 10.1001/jamanetworkopen.2024.15842 (PMC11177167; doi:10.1001/jamanetworkopen.2024.15842)
Supplement: Supplement 2. — eTable 1. Fidelity ratings for each treatment eTable 2. Differences between those with and without missing posttreatment or follow-up eTable 3. Descriptive statistics for primary and secondary outcomes eTable 4. Full results from moderation analyses on change in pain severity [file jamanetwopen-e2415842-s002.pdf]

Supplementary Online Content

Yarns BC, Jackson NJ, Alas A, Melrose RJ, Lumley MA, Sultzer DL. Emotional awareness and expression therapy or cognitive behavioral therapy for chronic pain in older veterans: a randomized clinical trial. *JAMA Netw Open*. 2024;7(6):e2415842. doi:10.1001/jamanetworkopen.2024.15842

eTable 1. Fidelity ratings for each treatment

eTable 2. Differences between those with and without missing posttreatment or follow-up

eTable 3. Descriptive statistics for primary and secondary outcomes

eTable 4. Full results from moderation analyses on change in pain severity

This supplementary material has been provided by the authors to give readers additional information about their work.

© 2024 Yarns BC et al. *JAMA Network Open*.

eTable 1. Fidelity ratings for each treatment

	EAET	CBT
Average fidelity rating (out of 34), mean (SD) range	33.45 (0.52) 32-34	33.15 (0.50) 32-34
Abbreviations: CBT, cognitive-behavioral therapy; EAET, emotional awareness and expression therapy		

© 2024 Yarns BC et al. *JAMA Network Open*.

eTable 2. Differences between those with and without missing posttreatment or follow-up

Characteristic	No. (%)		P
	Complete Case (n = 104)	Missing at Posttreatment or 6-month follow-up (n = 22)	
**Treatment Condition**			
CBT	50 (48)	10 (45)	0.99
EAET	54 (52)	12 (55)	
**Demographic characteristics**			
Age, mean (SD), y	71.7 (5.7)	73.0 (7.1)	0.45
Women	9 (9)	1 (5)	0.99
Men	95 (91)	21 (95)	
Race			0.27
American Indian or Alaska Native	0 (0)	1 (5)	
Asian/Pacific Islander	0 (0)	0 (0)	
Black (not of Hispanic origin)	58 (56)	11 (50)	
White (not of Hispanic origin)	31 (30)	8 (36)	
Multiracial or unknown	15 (14)	2 (9)	
Hispanic ethnicity	7 (7)	1 (5)	0.99
Marital status			0.76
Married/partnered	36 (35)	6 (27)	
Divorced/separated	41 (39)	9 (41)	
Never married or other	27 (26)	7 (32)	
Education			0.06
High school graduate or less	22 (21)	2 (9)	
Some college	46 (44)	16 (73)	
College graduate or more	36 (35)	4 (18)	
**Pain-related characteristics**			
Pain location			
Back	99 (95)	22 (100)	0.59
Neck	66 (63)	11 (50)	0.34
Leg	84 (81)	18 (82)	0.99
Pelvic or groin	38 (37)	10 (45)	0.47
Temporomandibular joint disorders	19 (18)	2 (9)	0.37
Fibromyalgia	3 (3)	2 (9)	0.21
Tension headaches	30 (29)	4 (18)	0.43
Pain duration, mean (SD), y	22.4 (17.9)	27.7 (16.7)	0.18
Prescribed opioids at baseline	13 (12)	1 (5)	0.46
**Other clinical characteristics**			
Any psychiatric diagnosis	74 (71)	13 (59)	0.31
VA service-connected for PTSD	65 (62)	14 (64)	0.99
Number of non-pain chronic medical conditions, mean (SD)	4.98 (2.45)	4.91 (2.41)	0.90

© 2024 Yarns BC et al. *JAMA Network Open*.

Number of prescription medications, mean (SD)	9.68 (4.80)	9.41 (5.00)	0.82
Mini-Mental State Examination score out of 30, mean (SD)	28.7 (1.4)	28.5 (1.4)	0.72
**Baseline outcome measure scores**	**Mean (SD)**		
BPI pain severity[a]	6.16 (1.77)	6.36 (1.94)	0.66
PROMIS Anxiety Short Form 7a[b]	19.4 (6.8)	19.3 (8.6)	0.96
PROMIS Depression Short Form 8a[c]	20.5 (7.9)	20.0 (9.1)	0.78
PROMIS Fatigue Short Form 7a[d]	20.9 (5.2)	22.1 (6.1)	0.37
NIH Toolbox General Life Satisfaction Fixed Form B[e]	14.9 (4.2)	14.7 (4.9)	0.88
PROMIS Pain Interference Short Form 8a[f]	28.4 (7.7)	29.8 (7.1)	0.42
PROMIS Sleep Disturbance Short Form 8a[g]	24.4 (7.9)	23.7 (9.4)	0.74
PCL-5[h]	26.6 (18.2)	30.5 (21.6)	0.48

Abbreviations: BPI, Brief Pain Inventory; CBT, cognitive-behavioral therapy; EAET, emotional awareness and expression therapy; PCL-5, PTSD Checklist for DSM-5; PROMIS, Patient Reported Outcomes Institute Measurement System.

[a]The primary outcome was the average of the four pain severity items of the BPI,[46] which measure current pain and worst, least, and average pain over the last 7 days, each on a 0-10 scale.

[b]The PROMIS Anxiety Short Form 7a[48] includes 7 items assessing anxiety symptoms over the last 7 days, each rated 1-5 for total scores ranging from 7 to 35; higher scores indicate greater anxiety.

[c]The PROMIS Depression Short Form 8a[48] includes 8 items assessing depressive symptoms over the last 7 days, each rated 1-5 for total scores ranging from 8-40; higher scores indicate greater depression.

[d]The PROMIS Fatigue Short Form 7a[49] includes 7 items assessing symptoms of fatigue over the last 7 days, each rated 1-5 for total scores ranging from 7 to 35; higher scores indicate greater fatigue.

[e]The NIH Toolbox General Life Satisfaction Fixed Form B[50] includes 5 items assessing the degree to which participants agree or disagree with statements about life satisfaction; each item is 1-5 for total scores between 5 and 25, and higher scores indicate greater life satisfaction.

[f]The PROMIS Pain Interference Short Form 8a[51] includes 8 items assessing pain's interference with mood and activities over the last 7 days, each rated 1-5 for total scores ranging from 8-40; higher scores indicate greater pain interference.

[g]The PROMIS Sleep Disturbance Short Form 8a[52] includes 8 items assessing sleep disturbance over the last 7 days, each rated 1-5 for total scores ranging from 8-40; higher scores indicate greater sleep disturbance.

[h]The PCL-5[55] includes 20 items on post-traumatic stress disorder symptoms related to the most stressful experience in life, each rated 0-4 for total scores between 0 and 80. Sample sizes for the PCL-5 are Complete Case, n=81; Missing Posttreatment or 6-month follow-up, n=18.

© 2024 Yarns BC et al. *JAMA Network Open*.

eTable 3. Descriptive statistics for primary and secondary outcomes

	Baseline	Post-treatment	6-month follow-up
Primary Outcomes			
**BPI pain severity**			
[1]CBT, mean (SD)	6.23 (1.63)	5.62 (1.85)	5.95 (1.75)
[2]EAET, mean (SD)	5.97 (1.98)	3.70 (1.96)	4.61 (2.09)
**At least 30% Pain Reduction**			
[1]CBT, (No.) %		(9) 17%	(7) 14%
[2]EAET, (No.) %		(36) 63%	(22) 41%
**At least 50% Pain Reduction**			
[1]CBT, (No.) %		(4) 7%	(2) 4%
[2]EAET, (No.) %		(20) 35%	(9) 17%
**At least 70% Pain Reduction**			
[1]CBT, (No.) %		(1) 2%	(1) 2%
[2]EAET, (No.) %		(7) 12%	(4) 7%
Secondary Outcomes			
**PROMIS Anxiety Short Form 7a**			
[1]CBT, mean (SD)	19.7 (7.3)	18.9 (6.8)	18.4 (7.4)
[2]EAET, mean (SD)	19.0 (7.0)	15.8 (5.7)	15.8 (6.2)
**PROMIS Depression Short Form 8a**			
[1]CBT, mean (SD)	20.3 (8.5)	18.1 (7.2)	19.3 (7.3)
[2]EAET, mean (SD)	20.6 (7.7)	15.4 (6.8)	17.1 (7.3)
**PROMIS Fatigue Short Form 7a**			
[1]CBT, mean (SD)	21.4 (5.4)	20.1 (6.2)	20.1 (5.1)
[2]EAET, mean (SD)	20.8 (5.4)	18.5 (5.5)	19.1 (5.3)
**NIH Toolbox General Life Satisfaction Fixed Form B**			
[1]CBT, mean (SD)	14.6 (4.1)	15.0 (3.5)	14.6 (3.7)
[2]EAET, mean (SD)	15.1 (4.4)	17.1 (4.5)	16.5 (4.1)
**PROMIS Pain Interference Short Form 8a**			
[1]CBT, mean (SD)	29.6 (7.0)	25.6 (8.1)	26.3 (7.9)
[2]EAET, mean (SD)	27.7 (8.1)	22.0 (8.5)	24.6 (8.4)
**PROMIS Sleep Disturbance Short Form 8a**			
[1]CBT, mean (SD)	24.0 (8.2)	23.3 (8.6)	24.2 (8.1)
[2]EAET, mean (SD)	24.6 (8.2)	21.3 (8.3)	21.4 (7.3)
**Patient Global Impression of Change**[a]			
[1]CBT, mean (SD)		3.2 (1.4)	2.8 (1.5)
[2]EAET, mean (SD)		4.7 (1.7)	4.0 (1.6)
**PCL-5**[b]			
[3]CBT, mean (SD)	25.5 (18.6)	24.5 (19.5)	24.1 (19.0)
[4]EAET, mean (SD)	28.9 (19.1)	23.7 (14.5)	24.6 (16.4)
**Satisfaction with Therapy**[c]			
[1]CBT, mean (SD)		23.6 (4.2)	

© 2024 Yarns BC et al. *JAMA Network Open.*

[2]EAET, mean (SD)		24.6 (5.0)	
**Satisfaction with Therapist**[d]			
[1]CBT, mean (SD)		25.6 (4.0)	
[2]EAET, mean (SD)		25.7 (4.7)	
**Global Satisfaction Score**[e]			
[1]CBT, mean (SD)		4.00 (0.54)	
[2]EAET, mean (SD)		4.28 (0.73)	

[1]Baseline N=60; Post-Treatment N=54; 6-Month Follow-Up N=50
[2]Baseline N=66; Post-Treatment N=57; 6-Month Follow-Up N=54
[3]Baseline N=47; Post-Treatment N=41; 6-Month Follow-Up N=38
[4]Baseline N=52; Post-Treatment N=44; 6-Month Follow-Up N=43

[a]Patient Global Impression of Change[53] is a single item rated 1-7 assessing change in activity limitations, symptoms, emotions, and quality of life since beginning treatment; higher scores indicate greater improvement.
[b]PCL-5, PTSD Checklist for DSM-5
[c]Satisfaction with Therapy subscale of the Satisfaction with Therapy and Therapist Scale-Revised (STTS-R)[54] includes 6 items rated 1-5 for possible scores ranging from 6 to 30; higher scores indicate better satisfaction.
[d]Satisfaction with Therapist subscale of the STTS-R[54] includes 6 items rated 1-5 for possible scores ranging from 6 to 30; higher scores indicate better satisfaction.
[e]Single item of the STTS-R[54] rated 1-5; higher scores indicate better satisfaction.

eTable 4. Full results from moderation analyses on change in pain severity

	EAET	CBT	EAET vs CBT Comparison	
	**EAET** Estimate (95% CI)	**CBT** Estimate (95% CI)	**Difference** Estimate (95% CI)	**P**
**Interaction with PROMIS Depression[a]**				
Change from baseline to posttreatment in pain severity for ***above vs below median depression***	**-1.42 (-2.49, -0.36)	.13 (-0.48, 0.73)	-1.55 (-2.73, -0.37)	0.01
Change from baseline to posttreatment in pain severity for ***1 SD increase in depression***	**-0.79 (-1.25, -0.32)	0.05 (-0.25, 0.34)	-0.83 (-1.32, -0.35)	<.001
**Interaction with PROMIS Anxiety[a]**				
Change from baseline to posttreatment in pain severity for ***above vs below median anxiety***	***-1.44 (-2.12, -0.76)	0.09 (-0.60, 0.78)	-1.53 (-2.19, -0.88)	<.001
Change from baseline to posttreatment in pain severity for ***1 SD increase in anxiety***	***-0.73 (-1.10, -0.36)	0.06 (-0.28, 0.40)	-0.80 (-1.19, -0.40)	<.001
**Interaction with PCL-5 Score[b]**				
Change from baseline to posttreatment in pain severity for ***above vs below median PTSD***	*-1.30 (-2.32, -0.28)	0.38 (-0.40, 1.17)	-1.69 (-2.95, -0.42)	0.009
Change from baseline to posttreatment in pain severity for ***1 SD increase in PTSD***	-0.35 (-0.78, 0.08)	0.13 (-0.49, 0.77)	-0.49 (-1.19, 0.22)	0.18

Abbreviations: 95% CI, 95% Confidence Interval; CBT, cognitive-behavioral therapy; EAET, emotional awareness and expression therapy; [a]PROMIS, Patient Reported Outcomes Institute Measurement System; [b]PCL-5, PTSD Checklist for DSM-5 (Sample sizes for the PCL-5 are EAET, n=52; CBT, n=47).
*p<.05; **p<.01; ***p<.001.

© 2024 Yarns BC et al. *JAMA Network Open*.
