# Supplementary material for: Emotional Awareness and Expression Therapy vs Cognitive Behavioral Therapy for Chronic Pain in Older Veterans: A Randomized Clinical Trial
Source: JAMA Netw Open. 2024 Jun 13;7(6):e2415842. doi: 10.1001/jamanetworkopen.2024.15842 (PMC11177167; doi:10.1001/jamanetworkopen.2024.15842)
Supplement: Supplement 3. — Data Sharing Statement [file jamanetwopen-e2415842-s003.pdf]

Data Sharing Statement

Yarns. Emotional Awareness and Expression Therapy vs Cognitive Behavioral Therapy for Chronic Pain in Older Veterans. *JAMA Netw Open*. Published June 13, 2024. doi:10.1001/jamanetworkopen.2024.15842

Data
Data available: Yes
Data types: Deidentified participant data
How to access data: To request consideration for access to data, please contact Alexander Alas, Study Coordinator, via email: alexander.alas@va.gov.
When available: With publication

Supporting Documents
Document types: None

Additional Information
Who can access the data: Researchers whose proposed use of the data has been approved.
Types of analyses: For pre-planned analyses and meta-analyses (if applicable)
Mechanisms of data availability: After approval of a proposal
